# Differences in photosystem II activity and carbon allocation during photomixotrophic growth in distinct wild‐type strains of *Synechocystis* sp. PCC 6803

**DOI:** 10.1111/tpj.70683

**Published:** 2026-01-24

**Authors:** Tuomas Huokko, Emil Sporre, Bradley Koch, Priyanka Pradeep Patil, Laura Wey, Lauri Nikkanen, Pornpan Napaumpaiporn, Olli Virtanen, Michal Hubácek, Natalia Kulik, Josef Komenda, Elton Hudson, Imre Vass, Yagut Allahverdiyeva

**Affiliations:** ^1^ Molecular Plant Biology, Department of Life Technologies University of Turku Turku Finland; ^2^ Department of Protein Science, Science for Life Laboratory KTH‐Royal Institute of Technology Stockholm Sweden; ^3^ University of Helsinki Helsinki Finland; ^4^ Institute of Plant Biology HUN‐REN Biological Research Center of HAS Szeged Hungary; ^5^ Doctoral School of Biology, Faculty of Science and Informatics University of Szeged Szeged Hungary; ^6^ Institute of Microbiology Centre Algatech Třeboň Czech Republic

**Keywords:** photomixotrophy, PSII, photosynthetic electron transport, carbon allocation, *Synechocystis* sp. PCC 6803

## Abstract

The regulation of photosynthetic electron transport during photomixotrophic growth in cyanobacteria remains incompletely understood. In this study, we characterized four wild‐type strains (WT 1–4) of *Synechocystis* sp. PCC 6803 and observed distinct strain‐specific differences in photosystem II (PSII) function under photomixotrophic conditions. Specifically, WT 1 and WT 2 exhibited near‐complete inhibition of electron transfer from Q_A_^−^ to Q_B_ following approximately 3 days of glucose supplementation, possibly mediated by binding of the small PSII‐associated protein, Psb28‐2, and resulting in a metabolic shift toward photoheterotrophy. Observed electron transport blockage was associated with changes in the abundances of various photosynthetic proteins. However, the structural integrity of both Photosystems appeared to be largely preserved. Such stabilization may be driven by a transient downregulation of linear electron transport to prevent overreduction of the electron transport chain under photomixotrophy. In contrast, WT 3 and WT 4 maintained photomixotrophic growth throughout the experiment but exhibited slower growth rates than WT 1 and WT 2. Although glucose uptake was slower in WT 1 and WT 2, both strains accumulated more glycogen than WT 3 and WT 4, suggesting divergent regulation of carbon allocation and storage metabolism. Together, these findings highlight the capacity of cyanobacterial strains to deploy distinct metabolic strategies to optimize photosynthetic function, carbon assimilation, and energy storage under photomixotrophic conditions.

## Significance Statement

Some cyanobacteria can use both light and organic carbon to support growth, but how they regulate photosynthesis under these conditions remains insufficiently understood. In this study, we show that wild‐type strains of *Synechocystis* sp. PCC 6803 can adopt distinct physiological strategies affecting PSII performance and carbon storage. This work reveals hidden diversity in how cyanobacteria adapt to combined carbon sources changing energy input, with implications for improving photosynthetic efficiency and designing robust strains for biotechnological applications.

## INTRODUCTION

Cyanobacteria perform oxygenic photosynthesis and contribute substantially to the global carbon cycle (Field et al., 1998). Oxygenic photosynthesis is essential for sustaining life, as it maintains atmospheric oxygen levels and serves as the primary source of energy and organic materials for most living organisms. In photosynthetic organisms, the thylakoid membranes host the light‐dependent electron transfer reactions of photosynthesis. These membranes contain key protein complexes including photosystem I (PSI), photosystem II (PSII), cytochrome (Cyt) b6f, and ATP synthase, which collectively drive photoautotrophic (PA) growth (Nikkanen et al., 2021).

Many cyanobacteria exhibit remarkable metabolic flexibility enabling growth not only through photoautotrophy but also under changing trophic conditions with varying energy and carbon availability. Photomixotrophy (PM), for instance, involves the simultaneous use of photosynthetic CO_2_ assimilation and exogenous organic carbon sources, such as glucose, sucrose, or acetate. By complementing photosynthesis with the uptake of organic carbon, PM promotes enhanced cell growth, leading to higher biomass yields and cell densities. This metabolic strategy is particularly relevant in nutrient‐rich and turbid aquatic environments, where limited light penetration coincides with organic matter (Muñoz‐Marín et al., 2024). PM is also crucial in microbial mats and biofilms, where sharp gradients of light and nutrients exist, making it an important factor in global biogeochemical cycles. Moreover, the versatility of photomixotrophic growth has significant potential for biotechnological applications aimed at improving biomass production in cyanobacteria (Matson & Atsumi, 2018).

The metabolic rearrangements that occur during PM in cyanobacteria are complex, as the cells rebalance the production between ATP and NADPH (Burnap et al., 2015; Zavřel et al., 2017). Cyanobacteria adjust their carbon fluxes to optimize both photosynthetic efficiency and organic carbon uptake. Under steady‐state ambient CO_2_ conditions, phosphoglucoisomerase (PGI) and the oxidative pentose phosphate pathway (OPPP) work synergistically to drive carbon metabolism, ultimately supporting the Calvin–Benson–Bassham (CBB) cycle for maximal CO_2_ fixation (Schulze et al., 2022). Conversely, in light‐limiting conditions, the regeneration of NADPH becomes essential, leading to an increased flux through the OPPP and requiring the action of transhydrogenase PntAB to balance the NADH/NADPH ratio in favor of NADPH production (Kämäräinen et al., 2017). The availability of CO_2_ also affects carbohydrate uptake rates, for example, with a molar uptake ratio of 6:1 for CO_2_ to glucose under elevated CO_2_ conditions and 2:1 under ambient conditions (Schulze et al., 2022; You et al., 2014). Proteomic analyses reveal that under photomixotrophic conditions, cyanobacteria downregulate components of the carbon concentrating mechanism (CCM), enhance nitrogen metabolism, and increase the abundance of phosphate transporters (Muth‐Pawlak et al., 2022). Additionally, shifts within the tricarboxylic acid (TCA) cycle promote alternative pathways for pyruvate biosynthesis (Cano et al., 2018). This dynamic adaptation of metabolic pathways enables cyanobacteria to store excess carbon, primarily in the form of glycogen, which provides a crucial buffering capacity for cellular metabolism (Cano et al., 2018; Ortega‐Martínez et al., 2023). Other potential storage compounds include polyhydroxybutyrate (PHB) (Price et al., 2020), cyanophycin (Watzer & Forchhammer, 2018), and polyphosphate granules (Gómez‐García et al., 2003), which serve as intracellular reserves of energy to support growth during fluctuations in external resources.

To support these metabolic adjustments, specific regulatory proteins are crucial for photomixotrophic growth in cyanobacteria. For example, PmgA, a serine–threonine kinase, is pivotal in adapting photosystem stoichiometry under high light conditions and regulating glycogen accumulation (Hihara et al., 1998; Sakuragi et al., 2006) in conjunction with the non‐coding RNA Ncr0700/PmgR1 (De Porcellinis et al., 2016). CP12, a regulatory protein of the CBB cycle that modulates carbon flux by thiol redox state‐dependent interactions with glyceraldehyde 3‐phosphate dehydrogenase and phosphoribulokinase to prevent futile cycling of carbon between the CBB cycle and OPPP (Wedel et al., 1997, e.g., Gurrieri et al., 2021), is essential for metabolic adjustment in cyanobacteria under diurnal, photomixotrophic conditions (Lucius et al., 2022). Cytochrome cM (CytM) also contributes to photomixotrophic growth by modulating photosynthetic activity, although its exact role remains uncertain (Solymosi et al., 2020).

In cyanobacteria, the majority of respiratory electron transfer occurs within the same protein complexes that facilitate photosynthetic electron transfer. Key redox‐active components, such as the plastoquinone (PQ) pool, the Cyt b_6_ *f* complex, and plastocyanin (PC) or Cyt c_6_, are shared between the photosynthetic and respiratory electron transport chains (ETCs) (Mullineaux, 2014). Under photomixotrophic conditions, the simultaneous processes of photosynthetic CO_2_ assimilation and the catabolism of exogenous organic carbon sources necessitate precise regulation of electron flow, and it is crucial to prevent an over‐reduced state in the shared components of the linear electron transport (LET) and respiratory ETCs. While respiratory activity generally increases under PM, the effects on photosynthesis can be more variable depending on the duration of photomixotrophic conditions (Haimovich‐Dayan et al., 2011; Lee et al., 2007; Solymosi et al., 2020; Takahashi et al., 2008).

Despite the ecological and biotechnological significance of PM, the molecular mechanisms that drive the transition of cyanobacteria from photoautotrophy to PM are not fully understood. This gap in knowledge particularly applies to the regulation of bioenergetic processes and the roles of various macromolecules in carbon storage. Multiple glucose‐tolerant strains of the cyanobacterium *Synechocystis* sp. PCC 6803 (hereafter referred to as *Synechocystis*) are classified as “wild‐type” (WT) (Koskinen et al., 2023). As previous studies have reported varying effects of PM on the photosynthetic activity and metabolism, we characterized four different *Synechocystis* WT strains under photomixotrophic conditions. Our investigation focused on the functionality of the photosynthetic apparatus including the oligomeric state and composition of photosynthetic complexes and the utilization of external carbohydrates. We also sequenced the genomes of all studied WT strains to reveal genetic modifications responsible for the observed differences. Our findings reveal that the photosynthetic machinery, particularly PSII activity, can be distinctly regulated under PM, and its extent varies between different *Synechocystis* WT strains leading to changes in the regulation and function of other parts of metabolism. These observations could inform the development of strategies aimed at engineering functional photosynthetic systems for biotechnological applications under photomixotrophic conditions.

## RESULTS

### Growth and photosynthetic characterization of studied WT strains under photomixotrophy

**Figure 1:**
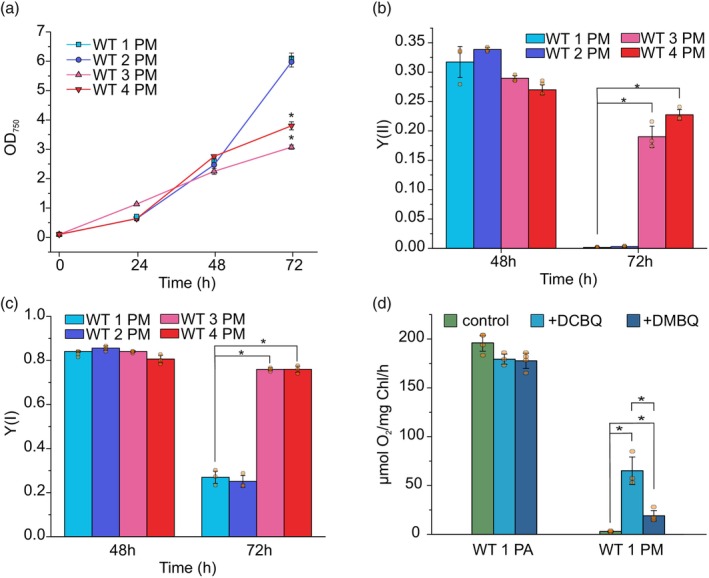
Growth and photosynthetic characterization of studied WT strains under photomixotrophy (PM). (a) growth of WT strains under photomixotrophy monitored by OD_750_, (b) the effective PSII yield, Y(II), after 48 h and 72 h of photomixotrophic growth, (c) the effective PSI yield, Y(I), after 48 h and 72 h of photomixotrophic growth, (d) the maximum gross O_2_ production rate in photoautotrophically (PA) or 72 h photomixotrophically (PM) grown WT 1, measured with and without the artificial electron acceptors DCBQ and DMBQ. Values are means ± SD; *n* = 3 biological replicates with individual data points shown as circles. Asterisks indicate statistically significant differences according to unpaired Student's *t*‐test (*P < 0.05).

In this study, we used four glucose‐tolerant WT strains of *Synechocystis*, originating from different labs (see Material and Methods), to thoroughly examine the effects of PM. As a first step, we assessed their growth over a 72 h period in the presence of 10 mm glucose. We also examined how the availability of inorganic carbon influences photomixotrophic growth by supplementing cultures daily with an additional 4.2 mm NaHCO_3_, with the initial growth medium pH adjusted to 7.5. During the first 48 h, all strains grew comparably; however, by 72 h, WT 1 and WT 2 continued to grow rapidly, whereas WT 3 and WT 4 showed significantly slower growth as measured by optical density (OD_750_) (Figure 1a). No significant differences in the cell number per OD_750_ were observed between these strains (Figure S1a), confirming variable growth abilities among the studied WT strains during PM. Notably, under PA conditions, all WTs demonstrated similar growth throughout the 72 h (Figure S1b), indicating that the growth advantage is specific to PM in the case of WTs 1 and 2. Moreover, comparable differences in growth between the strains were observed even without daily NaHCO_3_ supplementation (Figure S1c) and also when the medium pH was initially adjusted to 8.2 to increase the available HCO_3_^−^ concentration (Figure S1d). These findings suggest that the observed growth differences reflect distinct acclimation to PM rather than inorganic carbon limitation. Based on this, we conducted subsequent experiments under conditions of daily bicarbonate supplementation at pH 7.5, hereafter referred to simply as “photomixotrophy.”

To investigate the bioenergetic mechanisms underlying the variation in growth capacity during PM, we simultaneously monitored the activities of PSII and PSI. After 48 h, there were no substantial differences in the effective yields of PSII (Y(II)) (Figure 1b) and PSI (Y(I)) (Figure 1c) among the WT strains. However, by 72 h, Y(II) was nearly undetectable in WTs 1 and 2 (Figure 1b), with only approximately 30% of Y(I) remaining compared with the 48 h (Figure 1c). In contrast, WTs 3 and 4 retained approximately 66 and 84% of their Y(II), respectively (Figure 1b), and their Y(I) remained relatively unchanged (Figure 1c). Furthermore, LET was nearly undetectable in WT 1 after 72 h of PM and electron flow through P700 was predominantly maintained by cyclic electron transfer (CET) (e^−^ flux through PSI 8 ± 2 μmol e^−^/I/s), whereas WT 3 maintained approximately 60% LET and approximately 40% CET (e^−^ flux through PSI 30 ± 2 μmol e^−^/I/s) (Figure S1e,f). These results indicate that WTs 1 and 2, which exhibit fast growth capacity during 72 h of PM, have severely impaired LET due to strongly reduced PSII activity. In contrast, WTs 3 and 4, which show slower growth, maintain PSII activity throughout the 72 h. In line with this, WT 1 cultivated for 72 h under PM showed no transient P700 reduction and reoxidation upon switching on actinic red light illumination (Figure S1g), confirming the absence of electron flow from PSII. However, ferredoxin (Fd) redox kinetics were comparable in WTs 1 and 3 (Figure S1h), likely reflecting adjustments in the FNR/NADP^+^ pool level.

To better understand the bioenergetic mechanisms underlying diminished photosynthesis in WTs 1 and 2 after 72 h of PM, we monitored real‐time gas exchange fluxes in cells. Following 72 h of PM, dark respiration was only marginally increased in both WTs 1 and 2 compared with photoautotrophy (Figure S2). In contrast, the maximum gross O_2_ production rate dramatically decreased in both WTs 1 and 2 (dropping from 196.1 ± 8.5 μmol O_2_/mg Chl/h to 2.9 ± 0.6 μmol O_2_/mg Chl/h and from 197.3 ± 5.5 μmol O_2_/mg Chl/h to 5.9 ± 0.8 μmol O_2_/mg Chl/h, respectively) (Figure 1d, Figure S3). In line with this, CO_2_ uptake also decreased to undetectable levels (Figure S4). In the presence of the artificial electron acceptor 2,6‐dichloro‐1,4‐benzoquinone (DCBQ) which can accept electrons from Q_A_^−^ via binding the Q_B_ site (Kamada et al., 2023; Satoh et al., 1995) the maximum gross O_2_ production increased up to 22‐fold in photomixotrophic cells, reaching 65.1 ± 14.1 μmol O_2_/mg Chl/h in WT 1 and 83.2 ± 7.5 μmol O_2_/mg Chl/h in WT 2 (Figure 1d, Figure S3). Contrarily, in the presence of 2,6‐dimethoxybenzoquinone (DMBQ), which functions mainly at the level of the PQ pool (Graan & Ort, 1986; Satoh et al., 1995), the recovery of O_2_ production rates for photomixotrophically grown WT 1 (19.0 ± 5.3 μmol O_2_/mg Chl/h) and WT 2 (22.4 ± 4.0 μmol O_2_/mg Chl/h) (Figure 1d, Figure S3) were almost 70% lower compared with those with DCBQ. The maximum gross O_2_ production rates were not considerably affected by the addition of DCBQ or DMBQ under photoautotrophy. These results indicate that during PM, the primary hindrance for photosynthetic electron flow in WT 1 and WT 2 results from electron transfer within PSII from Q_A_^−^ to Q_B_.

### Monitoring Q_A_ ^−^ reoxidation in different WT strains during photomixotrophic growth

**Figure 2:**
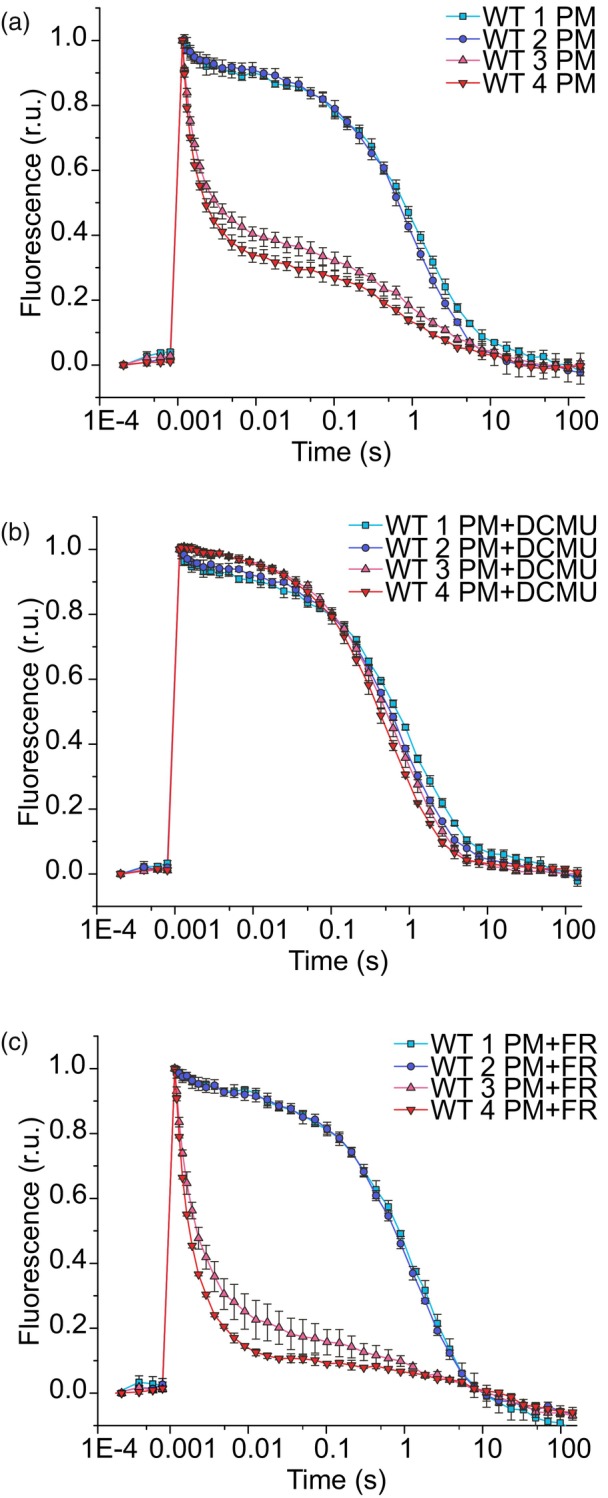
Relaxation of flash‐induced fluorescence yield in WT cells grown for 72 h under photomixotrophy (PM) (a) without DCMU supplementation, (b) with DCMU supplementation, and (c) with far‐red (FR) light pre‐illumination. Values are means ± SD; *n* = 3 biological replicates.

Next, we monitored the redox kinetics of the PSII primary electron acceptor Q_A_ by applying a single‐turnover saturating flash to dark‐adapted cells every 24 h for three consecutive days under PM. All WT strains demonstrated similar chlorophyll (Chl) fluorescence relaxation for up to 48 h of photomixotrophic growth compared with photoautotrophy (Figure S5). However, after 72 h of PM, fluorescence relaxation slowed strikingly in WTs 1 and 2, whereas WTs 3 and 4 showed only moderate changes in decay kinetics compared with earlier time points (Figure 2a).

To investigate the status of the PSII donor side, we monitored Q_A_^−^ reoxidation in the presence of 3‐(3,4‐dichlorophenyl)‐1,1‐dimethylurea (DCMU), which binds to the Q_B_ binding site, blocking electron transfer from Q_A_^−^ to Q_B_ and thus allows for the monitoring of charge recombination between Q_A_^−^ and the components of the oxygen‐evolving complex (OEC). Up to 24 h of PM, the Q_A_^−^ reoxidation kinetics in the presence of DCMU were similar in all WT strains (Figure S6b). However, after 48 h and increasingly after 72 h of PM, WTs 1 and 2, but not WT 3 and 4, demonstrated a small distinctive fast phase of decay even in the presence of DCMU (Figure 2b, Figure S6c).

To investigate the reoxidation of Q_A_^−^ in WT strains with varying PSII functionality during PM, we selected WTs 1 and 3 as representative strains of WTs 1/2 and 3/4, respectively, and further analyzed their fluorescence decay kinetics. In PSII centers with functional donor and acceptor side electron transfer, the decay of flash‐induced fluorescence consists of three main phases (Deák et al., 2014; Vass et al., 1999). The fast phase corresponds to the oxidation of Q_A_^−^ by Q_B_ (or Q_B_^−^), where the Q_B_ site has bound PQ when the flash is fired (time constant (*τ*)_1_ ~ 300–500 μs); the middle phase corresponds to the oxidation of Q_A_^−^ by PQ which binds to the Q_B_ site after the flash (*τ*_2_ ~ 5–15 ms); and the slow phase results from the recombination of the electron on Q_A_Q_B_^−^, via the Q_A_^−^Q_B_ ↔ Q_A_Q_B_^−^ charge equilibrium, with the oxidized S2 (or S3) state of OEC (*τ*_3_ ~ 10–20 s). Under photoautotrophy, both WTs 1 and 3 demonstrated similar Q_A_^−^ reoxidation kinetics typically associated with a functional PSII (Table 1). After 72 h of PM, WT 1 showed clear increase of time constants of the fast (from *τ*_1_ = 309.3 ± 43.0 μs to *τ*_1_ = 545.3 ± 107.3 μs) and middle phases (from *τ*_2_ = 3.11 ± 0.99 ms to *τ*_2_ = 28.32 ± 23.55 ms), whereas the slow phase demonstrated considerable acceleration (from *τ*_3_ = 8.05 ± 0.92 s to *τ*_3_ = 1.05 ± 0.06 s) compared with photoautotrophy. In contrast to time constants, WT 1 showed a marked decrease in the relative amplitudes of the fast (*A*_1_) and middle phases (A_2_) (dropping from 73.1 ± 3.0% to 9.9 ± 1.9% and from 18.8 ± 3.2% to 5.9 ± 0.8%, respectively) but a striking increase in the relative amplitude of the slow phase (from *A*_3_ = 8.1 ± 0.3% to *A*_3_ = 84.3 ± 2.4%) indicating that around 84% of PSII centers performed back reactions in WT 1, whereas back reactions occurred only in 31% of PSII centers (*A*_3_ = 31.0 ± 2.3%) in WT 3 at this stage.

**Table 1:** Multicomponent deconvolution of the fluorescence relaxation curves presented in Figure 2, Figures S5 and S6

	Fast phase	Middle phase	Slow phase
	τ_1_(μs)/*A*_1_ (%)	τ_2_(ms)/A_2_ (%)	τ_3_(s)/*A*_3_ (%)
WT 1 PA	309.31 ± 43.00/73.1 ± 3.0	3.11 ± 0.99/18.8 ± 3.2	8.05 ± 0.92/8.1 ± 0.3
WT 1 PM 24 h	296.48 ± 31.22/69.4 ± 1.7	2.40 ± 0.47/21.7 ± 0.4	10.33 ± 0.99/8.0 ± 1.4
WT 1 PM 48 h	295.97 ± 25.16/70.8 ± 2.5	2.26 ± 0.26/21.6 ± 2.8	4.85 ± 0.42^#^/7.6 ± 0.2
WT 1 PM 72 h	545.30 ± 107.31^#^/9.9 ± 1.9^#^	28.32 ± 23.55/5.9 ± 0.8^#^	1.05 ± 0.06^#^/84.3 ± 2.4^#^
WT 1 PA + DCMU	764.18 ± 100.36/2.3 ± 0.2		0.53 ± 0.01/97.7 ± 0.2
WT 1 PM + DCMU 24 h	919.96 ± 306.04/2.2 ± 0.3		0.53 ± 0.01/97.8 ± 0.3
WT 1 PM + DCMU 48 h	734.72 ± 27.52/2.8 ± 0.5		0.61 ± 0.02^#^/97.2 ± 0.5
WT 1 PM + DCMU 72 h	1513.32 ± 718.22^#^/9.0 ± 0.7^#^		0.83 ± 0.03^#^/91.0 ±0.7^#^
WT 3 PA	273.50 ± 11.44/70.9 ± 1.8	2.14 ± 0.15/21.2 ± 1.7	6.70 ± 0.65/7.9 ± 0.2
WT 3 PM 24 h	275.60 ± 17.36/67.2 ± 0.4	2.28 ± 0.10/24.8 ± 0.5*^,#^	10.33 ± 1.51^#^/8.0 ± 0.4
WT 3 PM 48 h	364.61 ± 9.91*^,#^/66.3 ± 4.4	3.13 ± 0.25*^,#^/19.4 ± 3.1	3.57 ± 2.86*/14.3 ± 6.9
WT 3 PM 72 h	449.33 ± 72.98^#^/53.4 ± 2.0*^,#^	6.20 ± 1.61^#^/15.6 ± 1.3*^,#^	0.97 ± 0.17^#^/31.0 ± 2.3*^,#^
WT 3 PA + DCMU	785.02 ± 182.62/2.8 ± 0.2		0.59 ± 0.01*/97.2 ± 0.2*
WT 3 PM + DCMU 72 h	4793.33 ± 1775.79*^,#^/3.8 ± 0.8*		0.53 ± 0.05*/96.2 ± 0.8*

Without DCMU supplementation the kinetics were analyzed in terms of two exponential components (fast and middle phase) and one hyperbolic component (slow phase) whereas in the case of DCMU supplementation with one exponential component (fast phase) and one hyperbolic component (slow phase), as described in Materials and methods. *τ*_1_–*τ*_3_ are the time constants and *A*_1_–*A*_3_ are the relative amplitudes as a percentage of total variable yield. Values are means ± SD; *n* = 3 biological replicates. Asterisks indicate statistically significant differences according to unpaired Student's *t*‐test (*P < 0.05) between WTs 1 and 3 under the same conditions, hash symbols relative to the corresponding PA sample (WT 1 PA, WT 1 PA + DCMU, WT 3 PA, or WT 3 PA + DCMU).

PA, photoautotrophy; PM, photomixotrophy.

In the presence of DCMU the Q_A_^−^ reoxidation kinetics are dominated by a 0.5–0.6 s phase originating from S_2_Q_A_^−^ recombination, but there is additionally a small fast phase with a time constant of a few ms, which could result either from recombination between Q_A_^−^ and the oxidized TyrZ^+^ on the donor side of PSII, or from incomplete inhibition by DCMU (Chu et al., 1994; Deák et al., 2014; Vass et al., 1999). DCMU supplementation after 24 h or 48 h under PM did not noticeably change the relative amplitudes or time constants of the fast or slow phases in WT 1 when compared with PA cells (Table 1). However, after 72 h of PM in DCMU‐supplemented WT 3, the time constant of the fast phase was threefold slower (*τ*_1_ = 4793.33 ± 1775.79 μs), accompanied by a reduced relative amplitude (*A*_1_ = 3.8 ± 0.8%), compared with WT 1 (*τ*_1_ = 1513.32 ± 718.22 μs, *A*_1_ = 9.0 ± 0.7%). In WTs 1 and 3, the slow phase of fluorescence decay demonstrated comparable relative amplitudes, but the time constant was significantly faster in WT 3 (*τ*_3_ = 0.53 ± 0.05 s) than in WT 1 (*τ*_3_ = 0.83 ± 0.03 s), with WT 3 resembling PA cells in the presence of DCMU. The similarity in slow phase time constants in WT 1, both with and without DCMU after 72 h of PM (0.83 ± 0.03 s vs. 1.05 ± 0.06 s), suggests that in this strain electrons for back reactions originate primarily from Q_A_^−^ rather than Q_B_^−^.

To determine if inhibition of Q_A_^−^ to Q_B_ electron transport in WTs 1 and 2 after sustained PM is mainly due to a highly reduced PQ pool, we pre‐illuminated cells with strong far‐red (FR) light prior to conducting flash fluorescence measurements (Ermakova et al., 2016), as FR light preferentially excites PSI, thus oxidizing the PQ pool. After 24 h (Figure S7a) and 48 h of PM (Figure S7b), FR pre‐illumination accelerated fluorescence decay in WTs 1 and 2, suggesting that PQ pool oxidation increased the fraction of PSII centers capable of forward electron transfer. In contrast, after 72 h of PM, FR pre‐illumination had minimal effect on the kinetics of fluorescence decay in these WT strains compared with measurements without FR pre‐illumination (Figure 2c). However, in WTs 3 and 4, the pre‐illumination with FR caused clearly faster relaxation of fluorescence after 72 h in PM. These results suggest that the main factor causing the inhibition of electron transfer from Q_A_^−^ to Q_B_ in WTs 1 and 2 after 72 h in PM is not a highly reduced PQ pool.

**Figure 3:**
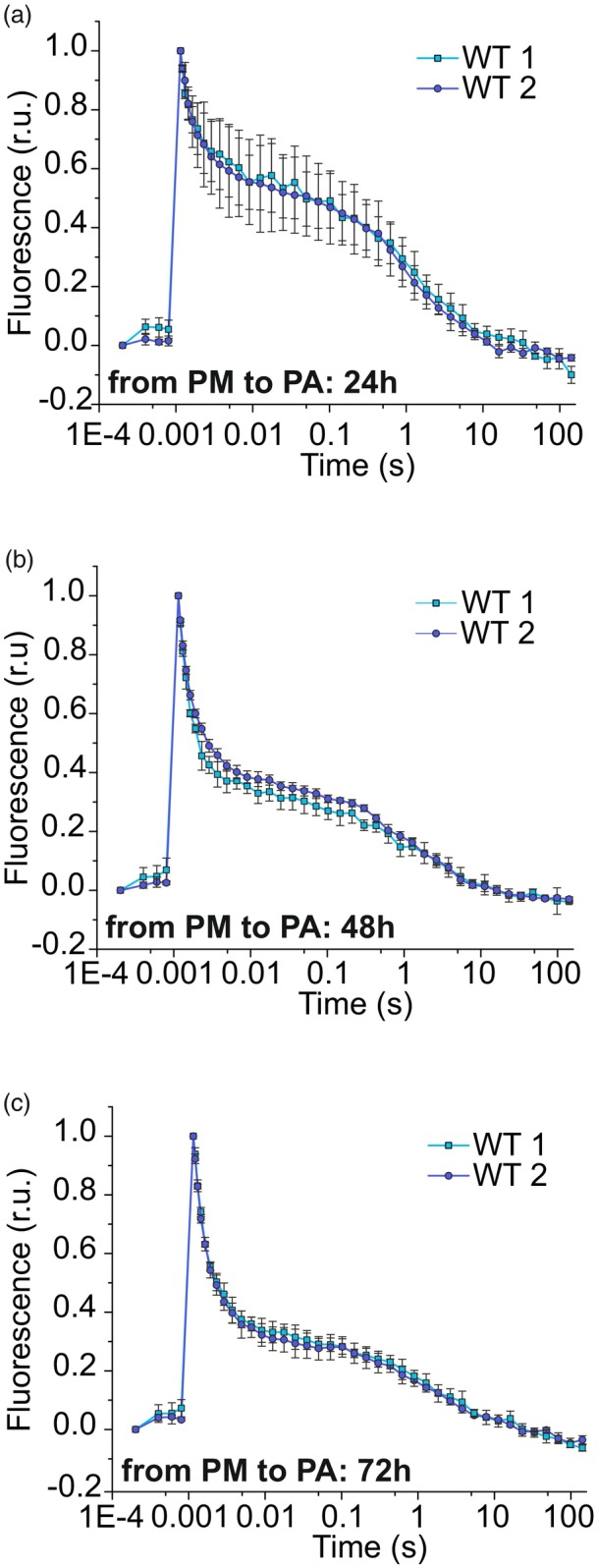
Re‐establishment of electron transfer within PSII in WT strains when trophy mode changes from photomixotrophy (PM) to photoautotrophy (PA). Relaxation of flash‐induced fluorescence yield in WTs 1 and 2 after (a) 24 h, (b) 48 h, and (c) 72 h of transferring cells from PM to PA conditions. Values are means ± SD; *n* = 3 biological replicates.

To assess the recovery of forward electron transfer from PSII, WTs 1 and 2 were transferred back to PA conditions after 72 h of growth under PM. After just 24 h, fluorescence decay accelerated substantially in both WTs 1 and 2 (Figure 3a) compared with PM (Figure 2a), indicating improved electron transfer from Q_A_^−^ to Q_B_ in a large fraction of PSII centers. This recovery continued progressively during photoautotrophy (Figure 3b,c), suggesting that the inhibition of electron transfer in PSII observed under PM is reversible and gradually relieved upon the transition to photoautotrophy.

### Proteomic changes induced by photomixotrophy

**Figure 4:**
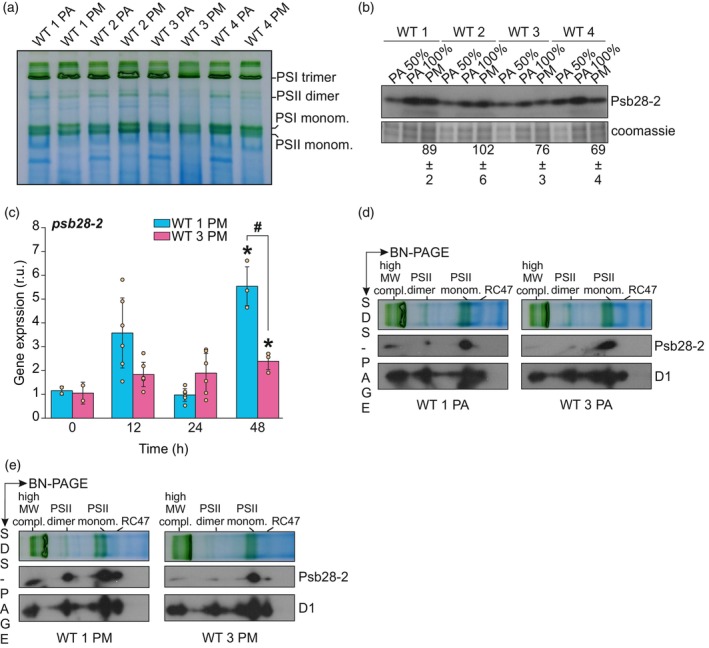
Analysis of thylakoid membrane protein complexes and Psb28‐2 accumulation in WT strains under photoautotrophic and photomixotrophic conditions. (a) BN‐PAGE analysis of thylakoid membranes isolated from cells grown under photoautotrophic (PA) and photomixotrophic (PM) conditions. (b) Immunoblot analysis of Psb28‐2 protein in WT strains grown under photoautotrophic (PA) and photomixotrophic (PM) conditions. Quantified values are relative to the corresponding PA sample, means ± SD; *n* = 3 biological replicates. Coomassie staining is represented as a loading control. (c) Quantitative reverse transcription (RT‐q) PCR analysis of *psb28‐2* transcript abundancies in WTs 1 and 3 under photomixotrophic conditions normalized to *rrn 16Sa* at the designated time points. Values are means ± SD; *n* = 3–5 biological replicates. Asterisk indicates statistically significant difference (*P < 0.05) compared with time point 0 h, hash sign between samples at each time point (^#^ *P* < 0.05) according to unpaired Student's *t*‐test. (d, e) 2D‐BN/SDS‐PAGE combined with immunoblotting using specific antibodies against Psb28‐2 and D1 proteins in (c) photoautotrophic and (d) photomixotrophic WTs 1 and 3. RC47: a monomeric PSII assembly intermediate that lacks the CP43 protein. Gel images and immunoblots are representatives of three biological replicates.

To examine the oligomeric states of photosynthetic complexes during PM, we performed blue native‐polyacrylamide gel electrophoresis (BN‐PAGE). The PSII dimer amount was reduced in all studied WT strains under PM, with a more prominent decrease in WTs 3 and 4 than in WTs 1 and 2 (Figure 4a). Similarly, PSI trimer levels were also reduced more in WTs 3 and 4 compared with WTs 1 and 2, although this difference was less pronounced than observed for the PSII dimer. These results indicate that WTs 1 and 2 maintain more of the photosystems in oligomeric forms under PM compared with WTs 3 and 4.

To investigate the broader proteomic differences between these strains, we performed a global label‐free tandem mass spectrometry (MS/MS) analysis using data‐independent analysis (DIA). We identified and quantified proteins from WTs 1 and 3, representing impaired and functional LET under 72 h of PM, respectively. Of these two WT strains, we quantified (with *P* ≤ 0.05) 1067 proteins with at least two peptides (Table S1). When the practical threshold of fold change (FC) was set to 1.5 (log2 1.5 = 0.58) for upregulated proteins and − 1.5 [−log2(1.5) = −0.58] for downregulated proteins, 363 proteins showed upregulation (Table S2) and 334 proteins downregulation in WT 1 compared with WT 3 under PM (Table S3).

Despite the differences in PSII dimer content (Figure 4a), no significant changes were observed in the accumulation levels of PSII core proteins between WTs 1 and 3 (Table S1), except for PsbF, a component of Cyt b559, which was lower in WT 1 compared with WT 3 (Table 2). Interestingly, the PSII‐associated protein Psb28‐2 (Slr1739), whose function remains poorly understood, was more abundant in WT 1 than in WT 3 (Table 2), whereas its homolog, the PSII assembly protein Psb28‐1 (Sll1398), showed no difference in abundance (Table S1). Additionally, WT 1, compared with WT 3, demonstrated decreased levels of several proteins participating in the photosynthetic electron transfer, including the major Rieske iron–sulfur protein of the Cyt b_6_f complex (PetC1), plastocyanin (PetE), Ferredoxin I (PetF), and the PSI subunits PsaC and PsaD (Table 2). Furthermore, both the large (RbcL) and small (RbcS) subunits of Rubisco, the bicarbonate transporter SbtA, CCM‐regulator SbtB, several carboxysome‐related proteins, and enzymes of CBB/OPP were less abundant in WT 1 compared with WT 3. This further supports the notion of active photosynthesis and CO_2_ fixation in WT 3 under PM. However, no changes were observed in the abundance of key enzymes of OPP (Zwf, GND) and PGI (PGI) shunts between the WT strains (Table S1). One of the key enzymes in glycogen biosynthesis, GlgC, was more abundant in WT 3, whereas GlgX and GlgP, which function in glycogen catabolism, were less accumulated compared with WT 1 (Table 2). Additionally, the levels of PhaA, E, and P, which are involved in PHB biosynthesis, were either not increased or even less accumulated in WT 3 compared with WT 1. In contrast, several phosphate transporters, along with enzymes involved in phosphate granule biosynthesis, were abundant in WT 3 compared with WT 1.

**Table 2:** Differential protein accumulation level in WT 1 versus WT 3 under photomixotrophy

	Protein	ORF	Log_2_FC (WT 1/WT 3)
PET	Psb28‐2	*slr1739*	1.4
	PsbF	*smr0006*	−2.7
	PetC1	*sll1316*	−1.4
	PetE (PC)	*sll0199*	−1.0
	PetF (Fd1)	*ssl0020*	−2.2
	PsaC	*ssl0563*	−1.2
	PsaD	*slr0737*	−3.0
Rubisco	RbcL	*slr0009*	−1.1
	RbcS	*slr0012*	−0.9
HCO_3_^−^ transport	SbtA	*slr1512*	−3.0
	SbtB	*slr1513*	−0.9
	CmpC	*slr0043*	1.8
CCM	CcmN	*sll1032*	−2.3
	CcmM	*sll1031*	−1.1
	CcmO	*slr0436*	−1.0
	CcmA	*sll0934*	0.9
CBB/OPPP	PGK	*slr0394*	−1.5
	Fba2	*sll0018*	−0.7
	Rpe	*sll0807*	1.4
	RpiA	*slr0194*	−0.6
Glycogen metabolism	GlgP	*sll1356*	1.0
	GlgX	*slr0237*	0.9
	GlgC	*slr1176*	−0.4
PHB biosynthesis	PhaE	*slr1829*	−0.3
	PhaA	*slr1993*	0.1
	PhaP	*ssl2501*	3.3
Phosphate transport and metabolism	PhoU	*slr0741*	−2.8
	PstB1	*sll0683*	−2.5
	PstB3	*slr1250*	−2.5
	PstB3	*slr1250*	−2.3
	PstB2	*sll0684*	−2.2
	PstB1	*sll0683*	−2.2
	PstB2	*sll0684*	−2.2
	PstB1	*sll0683*	−2.2
	PstA	*sll0682*	−1.2
	SphX	*sll0679*	−0.9
	Ppk	*sll0290*	−1.0

Proteins were quantified with DIA from cells grown in photomixotrophic conditions for 72 h.

PET, photosynthetic electron transfer; CCM, carbon concentrating mechanisms; CBB, Calvin–Benson‐Bassham cycle; OPPP, oxidative pentose phosphate pathway; PHB, polyhydroxybutyrate.

Given the differences in Psb28‐2 abundance observed in the proteomic analysis of photomixotrophic WTs 1 and 3, we determined Psb28‐2 levels in all four WTs using a specific antibody against it (Boehm et al., 2012). In WTs 1 and 2, Psb28‐2 levels remained relatively unchanged under PM (89% ± 2 in WT 1 and 102% ± 6 in WT 2, compared with PA), while they decreased in WTs 3 and 4 (76% ± 2 in WT 3 and 69% ± 4 in WT 2, compared with PA) (Figure 4b). These results suggest that WT strains with decreased PSII activity under PM, maintain higher Psb28‐2 compared with those with active photosynthesis.

We also analyzed the transcript levels of *psb28‐1* and *psb28‐2* in WT 1 and WT 3 during photomixotrophic growth using quantitative reverse transcription (RT‐q) PCR with specific primers (Figure S8, Table S4). The abundance of *psb28‐*1 transcripts did not differ between WT 1 and WT 3 after 48 h of PM (Figure S9). In contrast, at this time point, WT 1 exhibited a significantly higher *psb28‐2* transcript abundance than WT 3, even though both strains showed a significant increase compared with autotrophy (0 h), which was not observed at earlier time points (Figure 4c). This pattern is consistent with the corresponding protein abundances observed after 72 h under photomixotrophic conditions (Table 2, Figure 4b). To investigate the association of Psb28‐2 with different oligomeric forms of PSII in WTs 1 and 3, we performed 2D‐BN/SDS‐PAGE followed by immunoblotting using specific antibodies against Psb28‐2 and D1. Under PA conditions, Psb28‐2 was detected mainly in PSII monomers, with only minor amounts present in high‐molecular weight complexes containing PSII and very little associated with PSII dimers in either WT 1 or WT 3 relative to the PSII monomer (0.16 ± 0.05 and 0.11 ± 0.05, respectively; Figure 4d, Figure S10). Under photomixotrophic conditions, however, the distribution shifted: WT 1 showed a substantially higher proportion of Psb28‐2 in PSII dimers (0.62 ± 0.08 relative to monomers) compared with WT 3 (0.24 ± 0.07), accompanied by increased association with RC47 and high –MW complexes (Figure 4e, Figure S10). These data suggest greater association of Psb28‐2 with PSII dimers that show impaired electron transfer from Q_A_^−^ under PM.

**Figure 5:**
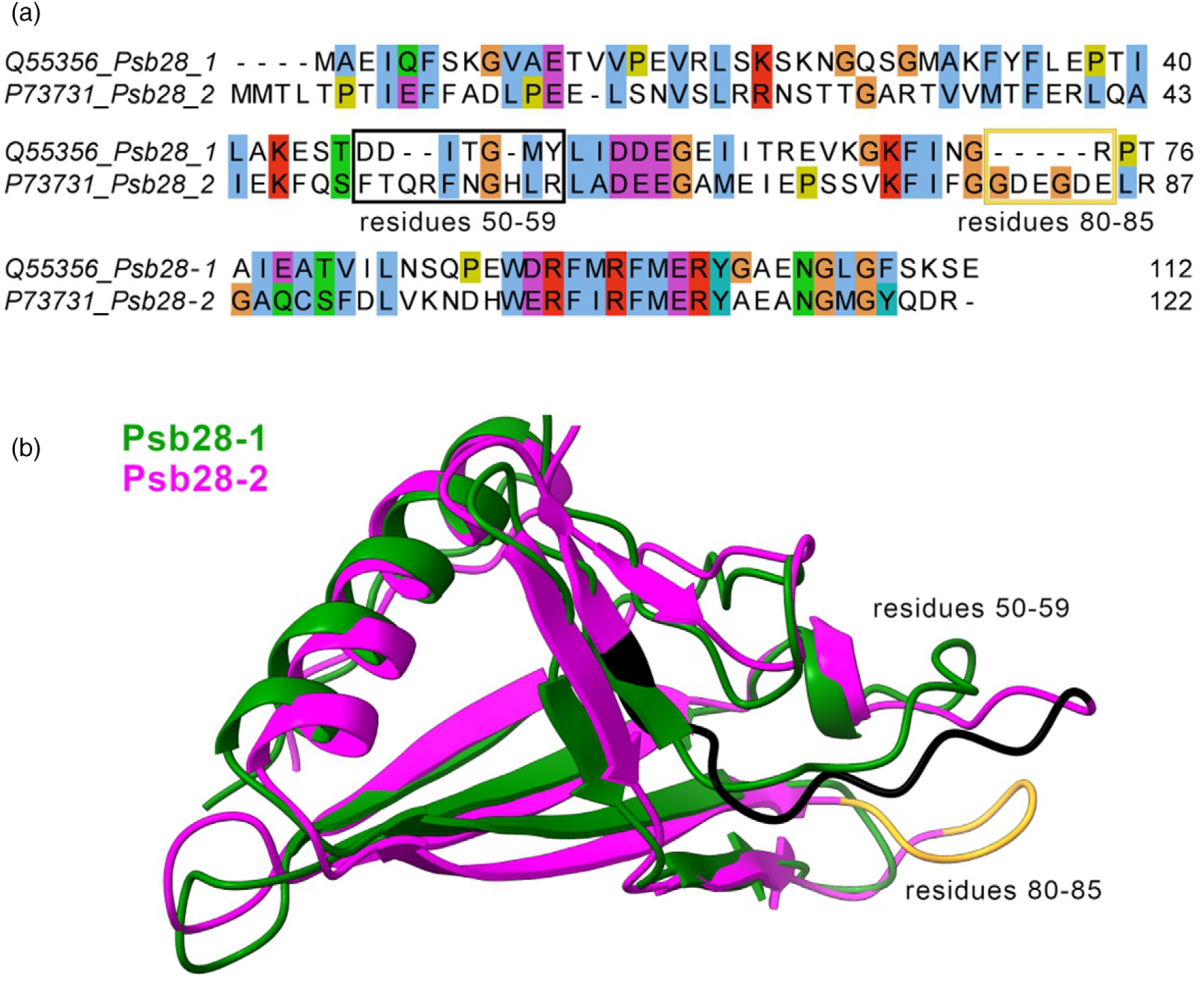
Comparison of Psb28‐1 and Psb28‐2 from *Synechocystis*. (a) Sequence alignment of Psb28‐1 and Psb28‐2 generated with ClustalX. Residues in Psb28‐2 that are frequently involved in interactions with D1 and D2 are highlighted with boxes. (b) Structural alignment of Psb28‐1 (green) and Psb28‐2 (magenta) models.

Structural and sequence analysis revealed several differences between Psb28‐1 and Psb28‐2, which may underline the distinct binding of Psb28‐2 to functional PSII complexes compared with Psb28‐1 (Figure 5). The most characteristic features of Psb28‐2 are a flexible, negatively charged loop (residues 80–85) and a variable region (residues 50–59) that differ from those in Psb28‐1.

### Utilization of glucose as carbon source in studied WT strains

**Figure 6:**
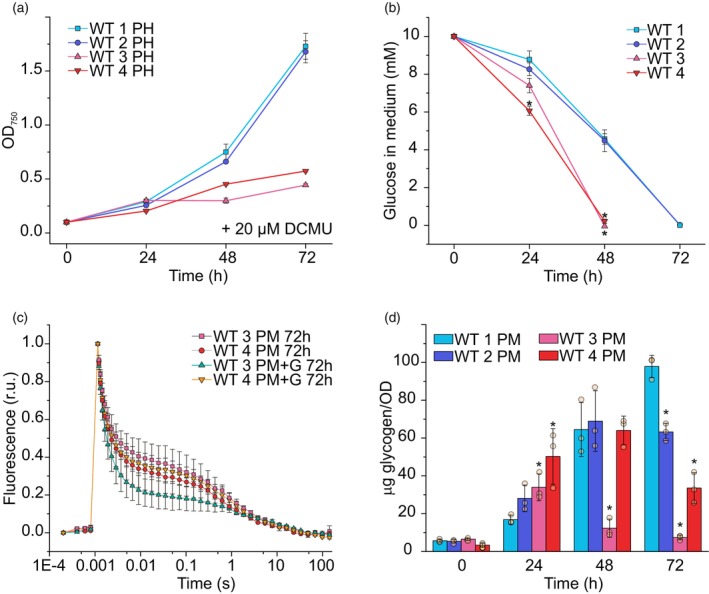
Glucose consumption and assimilation in the studied WT strains. (a) Photoheterotrophic (PH) growth monitored by OD_750_. (b) Glucose uptake from growth medium. (c) Flash‐induced fluorescence and subsequent decay in darkness in WTs 3 and 4 after 72 h of growth under photomixotrophy (PM) and after 72 h of growth under photomixotrophy with daily glucose supplementation (PM + G). (d) Intracellular glycogen accumulation during photomixotrophy (PM). Values are means ± SD; *n* = 3 biological replicates with individual data points shown as circles. Asterisks indicate statistically significant differences according to unpaired Student's *t*‐test (*P < 0.05) compared with WT 1 at each time point.

To assess whether differences in photosynthetic activity (Figures 1b,c and 2a, Table 1) and growth capacity (Figure 1a) of the studied WT strains under PM correlate with their glucose utilization ability, we monitored photoheterotrophic growth using glucose and DCMU. As DCMU inhibits electron transport from PSII, glucose becomes the only available carbon and energy source for cells. WTs 1 and 2 continued to grow throughout the experiment, whereas WT 3 and 4 stalled their growth after 48 h (Figure 6a), indicating reduced glucose assimilation capacity. Without DCMU supplementation, WTs 3 and 4 nearly depleted glucose within 48 h, while WTs 1 and 2 fully consumed the entire 10 mm glucose pool by 72 h (Figure 6b).

To exclude the possibility that glucose depletion at 48 h affects photosynthetic activity in WTs 3 and 4, we supplemented them daily with an additional 5 mm glucose, in addition to the initially supplied 10 mm. This had no significant effect on fluorescence kinetics (Figure 6c), indicating that the sustained electron transfer from Q_A_^−^ to Q_B_ in WTs 3 and 4 under PM is unrelated to faster glucose depletion. Furthermore, WTs 1 and 2 accumulated more intracellular glycogen during PM than WTs 3 and 4 (Figure 6d), suggesting that the slower growth of WTs 3 and 4 is not due to greater carbon storage as glycogen, despite their faster glucose uptake.

### Next‐generation sequencing (NGS) of studied WT strains

To investigate whether the differential function of PSII during PM is caused by genetic variation between studied WT strains, we sequenced their genomes using next‐generation sequencing (NGS). The genome of the WT Kazusa (Kaneko et al., 1996) was selected as a reference genome, and reads from WTs 1, 2, 3, and 4 were mapped to it. The length of the chromosome was 3 573 468 bp in WT 1, 3 573 467 bp in WT 2, 3 573 477 bp in WT 3, and 3 573 509 bp in WT 4 (https://www.ncbi.nlm.nih.gov/bioproject/?term=PRJNA1277488). These values closely match the WT Kazusa reference strain, which has a chromosome length of 3 573 470 bp (Kaneko et al., 1996), indicating no substantial differences, such as large deletions or insertions, in the studied WT genomes.

**Figure 7:**
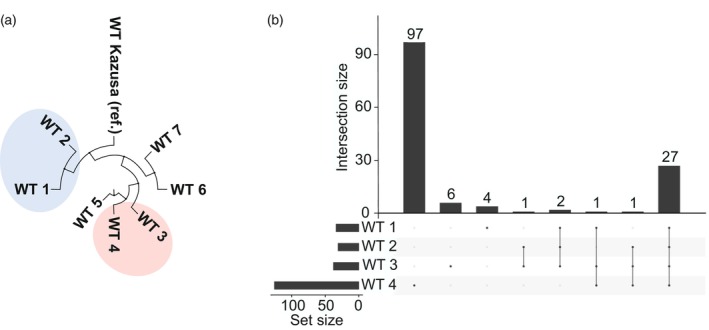
Genetic characterization of studied WT strains. (a) Phylogenetic tree of several WT strains. Highlighted parts contain the WT strains sequenced and used in measurements of this study. (b) The concordance graph of genes potentially affected by mutations in the coding region in each studied WT strain. In X‐axis “Set size” indicates how many individual genes were mutated in each strain and in Y‐axis “Intersection size” shows how many genes fell into the specific intersection. Dots under each column indicate which WT strains shared mutations in the same genes compared with reference strain WT Kazusa.

Compared with the WT Kazusa reference strain, WT 1, WT 2, WT 3, and WT 4 carried 315, 364, 480, and 496 any type of mutations, respectively (Tables S5, S6 and S8). To assess the genetic similarity among the WT strains, we constructed a phylogenetic tree based on the whole‐genome sequencing data, including three additional, previously sequenced *Synechocystis* WT strains (WT 5, WT 6, and WT 7) (https://www.ncbi.nlm.nih.gov/bioproject/?term=PRJNA1277488). This analysis showed that WTs 1 and 2 clustered closely with the WT Kazusa reference, whereas WTs 3 and 4 formed a distinct cluster that was more distant from the reference strain but closely related to each other (Figure 7a). This is in line with the physiological measurements observed under PM.

We next investigated mutations in coding regions that result in frameshift mutations, missense mutations, or premature stop codons. Across all studied WT strains, 27 mutated genes were shared (Figure 7b, Table S9). Additionally, WT 1 had four unique mutations, WT 3 had six mutations, and WT 4 carried 97 mutations. However, we did not identify any coding region mutations present in both WTs 3 and 4 but absent from WT 1 and WT 2 which could plausibly account for the observed differences in PSII activity among photomixotrophic conditions.

## DISCUSSION

PM provides a metabolic advantage by enabling organisms to utilize both photosynthetic CO_2_ assimilation and external organic carbon sources for growth. We investigated its effects on photosynthetic capacity and carbon metabolism during 72 h of PM on four *Synechocystis* WT strains, which were re‐sequenced to identify possible genetic changes underlying the observed phenotypic differences. As expected, PM enhanced the growth of all WT strains compared with photoautotrophy (Figure 1a, Figure S1b). Notably, despite their higher glucose uptake capacity (Figure 6b) and active PSII function (Figure 1b), WTs 3 and 4 did not outperform WTs 1 and 2 during either the early or later stage of photomixotrophic growth (Figure 1a). These unexpected differences in growth performance among the WT strains suggest that, beyond glucose uptake and PSII activity, the ability to efficiently store and manage carbon may play a key role in supporting photomixotrophic growth. In line with this, efficient carbon storage and metabolism are crucial to prevent the accumulation of toxic intermediates, like ADP‐glucose (Díaz‐Troya et al., 2020). Glycogen, the primary carbon storage molecule during PM (Nakajima et al., 2014; Schulze et al., 2022; Yoshikawa et al., 2013), facilitates maintaining metabolic balance by generating NADPH when a reducing equivalent is needed or serving as a sink for carbon and ATP during energy excess (Cantrell et al., 2023). Maintaining flux between glycogen and central carbon metabolism is vital for cellular fitness (Ortega‐Martínez et al., 2023) and glycogen‐deficient mutants exhibit rapid PSII inhibition under PM due to central metabolite accumulation (Ortega‐Martínez et al., 2024). However, this is not the case of photosynthetic inactivation in WTs 1 and 2 as they accumulated substantial amounts of intracellular glycogen during PM (Figure 6d).

After 48 h of PM, WTs 1 and 2 retained characteristics of photoheterotrophic cells with inactive PSII and exponential growth (Figure 1b,d 2a and 6a). Thus, it is likely that glycogen reserves in PM support NAPDH production for glycolysis (e.g., via the OPPP shunt) in WTs 1 and 2 at a high enough level, eliminating the need for CO_2_ fixation as an ATP sink (Figure S4) and sustaining fast growth even after glucose depletion. Carbon metabolism‐dependent control of CO_2_‐fixation is reasonable also from ecological point of view, as photoheterotrophy represents an important metabolic strategy among photosynthetic phytoflagellates in aquatic ecosystems (Wilken et al., 2014). In turn, WTs 3 and 4 possibly face inefficiencies in carbon allocation and glucose metabolism via heterotrophic metabolic pathways, which is suggested by their reduced growth under photoheterotrophic conditions (Figure 6a). In these strains, as glucose became depleted from the growth medium (Figure 6b), WT 4 began to degrade glycogen, while WT 3 showed limited glycogen accumulation during continued photomixotrophic growth (Figure 6d), neither of which were affected by extra additions of extracellular glucose (Figure 6c). This suggests that WT 3 in particular may experience an energy imbalance at 72 h of PM, possibly due to excess ATP, which could be mitigated through various mechanisms such as ATP consumption via CO_2_ fixation requiring active photosynthesis, engagement in futile cycles for example, glyceraldehyde 1,3‐bisphosphate cycling to hydrolyze excess ATP (Livingston et al., 2012), or by overflow metabolism involving the secretion of organic acids (Cano et al., 2018; Gründel et al., 2012). Another plausible mechanism for excess ATP dissipation in WT 3 is the formation of polyphosphate granules, which serve as ATP storage and accumulate under photomixotrophic conditions in cyanobacteria (Sebesta et al., 2024). This is supported by the accumulation of phosphate uptake systems and polyphosphate kinase (Ppk) (Table 2), in agreement with an earlier study by Muth‐Pawlak et al. (2022). Additionally, inorganic phosphate (P_i_) has been shown to inhibit glucose‐1‐phosphate adenylyltransferase (GlgC), the enzyme catalyzing the first step of glycogen synthesis (Iglesias et al., 1991; Lee et al., 2024). Together, these findings highlight the importance of metabolic flexibility and efficient carbon storage in cyanobacteria, particularly under PM, and how various background strains with only limited genetic variability (Figure 7) can exhibit markedly different responses to various sources of carbon.

While short‐term effects of PM on cyanobacterial photosynthetic capacity vary (Haimovich‐Dayan et al., 2011; Lee et al., 2007; Takahashi et al., 2008; Zilliges & Dau, 2016), prolonged exposure to photomixotrophic conditions appears to decrease photosynthetic efficiency in cyanobacteria (Solymosi et al., 2020) as well as in other photosynthetic organisms (Sun et al., 2023; Wilken et al., 2014). 72 h of PM led to a gradual decline in photosynthetic capacity in all WT strains compared with photoautotrophy, with PSII being the most affected (Figure 1b,c and Figure 2a). However, the extent of this decline varied drastically between strains. In WT 1, almost 84% of PSII centers performed back reactions from Q_A_^−^ to donor side components, mainly the S_2/3_ state of OEC, instead of performing forward electron transport to Q_B_ and the PQ pool (Figure 2a, Table 1). In contrast, only 30% of PSII centers in WT 3, which maintained active photosynthesis, performed back reactions under PM.

Furthermore, our findings suggest that temporarily halting photosynthesis during PM, most likely as a transient response, helps protect the integrity of the photosynthetic machinery. This is supported by both higher PSII dimer/monomer and PSI trimer/monomer ratios in WTs 1 and 2 with only marginal PSII activity (Figures 1d and 4a, Figure S3). Since PSII dimers have been demonstrated to be more biologically active than monomers (Nowaczyk et al., 2006), maintaining PSII in dimeric form would be beneficial for the cells. A higher amount of PSII in its dimeric form under PM could be partially maintained by disconnecting electron transfer from Q_A_^−^ to Q_B_ as this may protect PSII centers from irreversible photodamage. Decelerated electron transfer between Q_A_^−^ and Q_B_ results in longer‐lived Q_A_^−^, which reduces the binding affinity of the bicarbonate involved in Q_B_ protonation, to the non‐heme ferrous iron (Fe_2_^+^) in PSII (Cardona et al., 2012; Müh et al., 2012; Shevela et al., 2012). Consequently, this bicarbonate is released, increasing the likelihood of back reactions in PSII by upshifting the E_m_ of Q_A_/Q_A_^−^ (Brinkert et al., 2016; Fantuzzi et al., 2022). As more positive potential for Q_A_/Q_A_^−^ widens the energy gap between P680^+^/Q_A_^−^ and P680^+^/Pheo^−^, the shift favors the direct recombination of P680^+^/Q_A_^−^ over the back reaction via P680^+^/Pheo^−^ (Johnson et al., 1995). This mechanism is advantageous for cells, as it significantly reduces the generation of the chlorophyll triplet, ^3^P680, which can form toxic singlet oxygen (^1^O_2_) in the presence of O_2_. Despite strongly decreased electron transfer from Q_A_^−^ to Q_B_ during PM in WTs 1 and 2 (Figure 2a, Table 1) and reduced dimeric PSII content compared with PA conditions (Figure 4a), a significant portion of PSII retained a moderately functional OEC, as shown by partial rescue of gross O_2_ evolution with DCBQ (Figure 1d, Figure S3). This suggests that inhibiting forward electron transfer in this manner within PSII helps to regulate its activity with minimal disruption to its oligomeric forms and generally to the photosynthetic machinery when a more readily available carbon source is present. Upon returning to PA growth, electron transfer between Q_A_^−^ and Q_B_ gradually resumed (Figure 3), likely due to adjustments in cell metabolism.

A key question is what inhibits electron transfer from Q_A_^−^ to Q_B_ during continued PM. In cyanobacteria, maintaining the redox balance of the PQ pool is probably more critical than maximizing reducing power and energy storage in NADPH and ATP, most likely serving as a key survival strategy in dynamic open water environments characterized by fluctuating light and nutrient conditions (Milou Schuurmans et al., 2014). This appears to be the case also under PM (Solymosi et al., 2020). In the green alga *Chlamydomonas reinhardtii*, anoxic conditions lead to high reduction of the PQ pool through non‐photochemical inputs from stromal reductants (e.g., glycolysis), that inhibits electron transfer from Q_A_^−^ to Q_B_. This overreduction, nevertheless, can be alleviated, allowing partial restoration of electron flow (Nagy et al., 2018; Volgusheva et al., 2013; Volgusheva et al., 2016). However, the inhibition of electron transfer observed in *Synechocystis* WTs 1 and 2 after 48 h of PM is unlikely to occur via the same mechanism, as forced PQ pool oxidation with FR‐illumination had no effect on fluorescence decay kinetics at this stage (Figure 2c). In contrast, the same FR treatment accelerated fluorescence decay in WT 3 and 4 under similar conditions (Figure 2c), as well as in WTs 1 and 2 earlier during PM (Figure S7), when forward electron flow from Q_A_^−^ to Q_B_ was still evident (Figure S5). About 3.5‐fold higher gross O_2_ evolution was observed in WTs 1 and 2 with DCBQ, which replaces PQ at the Q_B_ site (Kamada et al., 2023; Satoh et al., 1995), compared with DMBQ, which accepts electrons downstream of PQH_2_ release (Graan & Ort, 1986; Satoh et al., 1995) (Figure 1d, Figure S3). Furthermore, virtually no O_2_ evolution was detected in photomixotrophic WT 1, while O_2_ evolution could be partially recovered by the addition of DCBQ as an artificial electron acceptor, but not with DMBQ (Figure 1d, Figure S3). Hence, we suggest that in WTs 1 and 2 the Q_B_ pocket of PSII undergoes structural changes under photomixotrophic conditions, largely preventing PQ‐binding/PQH_2_‐release while still allowing limited interaction with DCBQ. Further evidence for alterations in the Q_B_ pocket is provided by the slower kinetics of the middle phase in WT 1 compared with WT 3 after 72 h of photomixotrophic growth (Table 1). These findings align with studies of *Synechocystis* mutants with deletions in the sequence of the D–E loop of PSII reaction center protein D1 resulting in severely modified Q_B_ pockets, where DCBQ also supported significantly higher O_2_ evolution than DMBQ (Mulo et al., 1997; Mulo et al., 1998).

The alteration in the Q_B_ pocket that impedes electron transfer from Q_A_^−^ to Q_B_ during PM may stem from changes in the protein composition of the PSII complex. For example, the absence of PsbJ in cyanobacterial PSII results in long‐lived Q_A_^−^, obstructing electron flow to Q_B_ and reducing oxygen production (Regel et al., 2001). However, in the studied WT strains, no mutations leading to deletions (Tables S5–S9) or substantial changes in the abundance of PSII core subunits (Tables S1–S3) were identified. Additionally, spontaneous mutations in the PSII core proteins D1 and D2 can also impact electron transfer. In *Synechocystis*, replacing tyrosine 246 of D1 with alanine enhances the back reaction with the S2 state of the OEC (Forsman et al., 2019), while in *Chlamydomonas reinhardtii*, A250R and S264K substitutions in D1 disrupt the Q_B_ site hydrogen bond network, slowing Q_A_^−^ reoxidation and reducing O_2_ production (Antonacci et al., 2018). However, no such mutations were found in the D1 or D2 genes of the studied WT strains, except for a synonymous mutation in *psbA3*, common to all strains (Tables S5–S9).

It has been reported that Psb28‐2 protein abundance increases during the transition to PM, while Psb28‐1 abundance remains unchanged (Muth‐Pawlak et al., 2022). Consistent with this, Psb28‐2 levels were higher in WT 1 and WT 2 strains with impaired PSII under PM compared with those maintaining active photosynthesis (Figure 4b, Table 2) and this was observed also at the transcript level (Figure 4c), indicating transcriptional regulation. Importantly, in these conditions, the association of Psb28‐2 with PSII dimers was notably higher in WT1 with inactive electron transfer from Q_A_^−^ to Q_B_ than in WT3, which retained functional photosynthesis (Figure 4e, Figure S10), while no substantial difference was observed under photoautotrophy (Figure 4d, Figure S10). Psb28‐2, a homolog of Psb28‐1 in *Synechocystis*, is structurally similar to Psb28‐1, and it has been suggested that both proteins share the same binding region on PSII (Boehm et al., 2012). However, unlike Psb28‐1, which primarily binds to the RC47 complex, a PSII intermediate lacking CP43, Psb28‐2 is more abundant in the monomeric and dimeric forms of PSII (Bečková et al., 2017). This is in agreement with the function of Psb28‐1, a small soluble protein having a role in PSII assembly by binding to the cytoplasmic surface of the complex. During this process, it interacts with D1, D2, and CP47, inducing conformational changes in the Q_A_ and Q_B_ binding sites, as well as altering the coordination environment and the hydrogen bond network around the non‐heme iron (Xiao et al., 2021; Zabret et al., 2021). This interaction results in the replacement of the bicarbonate ligand of non‐heme iron with a glutamate residue, shifting the redox potential of the Q_A_ /Q_A_^−^ toward a more positive value and increasing the probability for direct recombination of the charge‐separated states (Brinkert et al., 2016). Such a shift may also suppress the charge recombination via the P680 + •/Pheo−• pathway, while enhancing recombination from Q_A_^−^ to Tyr_Z_^+^, thereby protecting PSII from oxidative damage during PSII assembly (Johnson et al., 1995; Xiao et al., 2021; Zabret et al., 2021).

We propose that under PM, Psb28‐2 binding to mature PSII (Figure 4e) may contribute to the inhibition of electron transfer from Q_A_^−^ to Q_B_. Unlike Psb28‐1, Psb28‐2 contains five additional negatively charged amino acid residues (DELRG) in a short loop at positions 83–87, as well as extra charged and aromatic residues at positions 53–59 (Figure 5). These residues may enable interactions with the mature PSII dimers, thus inhibiting electron transfer between Q_A_^−^ and Q_B_. However, considering the remaining PSII dimers and monomers in photomixotrophic WT 1 (Figure 4e), this interaction does not induce such detrimental changes to PSII stability. In contrast, the binding of Psb28‐1 during PSII assembly destabilizes the association of CP43, disrupts the Q_B_ binding pocket, and replaces the bicarbonate ligand of the non‐heme iron with glutamate (Xiao et al., 2021; Zabret et al., 2021).

These findings suggest that Psb28‐2 may play a specialized role in modulating PSII activity under PM by selectively inhibiting electron transfer from Q_A_^−^ to Q_B_ and maintaining PSII structural integrity. However, the observed inhibition of electron transfer in PSII may not be solely attributed to the binding of Psb28‐2 to PSII. Metabolic differences between the WT strains, especially the observed alterations in glycogen accumulation (Figure 6d), can also play a role in modulating photosynthetic electron transport, having downstream effects on photosystem activity. The slowing down of electron transfer from Q_A_^−^ to Q_B_ may also result from the exchange of bicarbonate at the non‐heme Fe with small carboxylic acids, which has been observed in *Chlamydomonas* under mixotrophic growth conditions (Roach et al., 2013). Furthermore, modulation of PSII activity might serve as a fine‐tuning mechanism regulated by intracellular energy levels or specific metabolites, like glycogen, linking photosynthetic performance to broader metabolic adjustments.

In summary, our findings show that closely related *Synechocystis* WT strains can employ fundamentally different strategies to regulate photosynthetic electron flow and carbon storage under mixotrophic conditions. This divergence highlights the metabolic flexibility within the species and its ecological significance in adapting to diverse environments. Furthermore, these insights could contribute to the development of strategies for optimizing carbon allocation in cyanobacteria for bioengineering applications.

## MATERIALS AND METHODS

### Strains and culture conditions

Glucose‐tolerant *Synechocystis* sp. PCC 6803 WT strains (WTs 1, 2, 3, and 4) used in this study originated from various laboratories. WT 1 originated from Aaron Kaplan's lab at The Hebrew University of Jerusalem, Israel, and was obtained from the University of Turku, Finland. WT 2 originated from Chris Howe's lab at the University of Cambridge, UK. WTs 3 and 4 were provided by Imre Vass at the Institute of Plant Biology, HUN‐REN Biological Research Center, Hungary; WT 3 originated from Peter Nixon's lab at Imperial College London, UK, while WT 4 originated from Kazuyoshi Murata's lab at the National Institute for Physiological Sciences, Japan.

Pre‐experimental cultures were grown in BG11 medium buffered with 20 mm HEPES‐NaOH (pH 7.5) or with 10 mm TES‐KOH (pH 8.2) under continuous white light of 50 μmol photons m^−2^ s^−1^ (PAR) at 30°C and air enriched with 3% CO_2_ with agitation of 150 rpm. PA experimental cultures were grown in BG11 medium buffered with 20 mm HEPES‐NaOH (pH 7.5) and photomixotrophic experimental cultures were grown in BG11 buffered with 20 mm HEPES‐NaOH (pH 7.5) or with 10 mm TES‐KOH (pH 8.2) supplemented with 10 mm d‐glucose (added at the beginning of experiment if not mentioned otherwise) and with or without daily 4.2 mm NaHCO₃ supplementation. Photoheterotrophic experimental cultures were grown in BG11 (pH 7.5) supplemented with 10 mm d‐glucose and 20 μM DCMU at the beginning of experiment as well as daily 4.2 mm NaHCO₃ addition.

All experimental cultures were started from pre‐experimental cultures by harvesting cells, resuspending them to fresh BG11 and inoculating cells to the starting OD_750_ = 0.1 and grown under continuous illumination of 50 μmol photons m^−2^ s^−1^ (PAR) at 30°C with agitation of 150 rpm in growth chambers with cool‐white light‐emitting diodes (AlgaeTron AG130 by PSI Instruments, Drásov, Czech Republic). OD_750_ was measured using the Genesys 10S UV–Vis spectrophotometer (Thermo Fisher Scientific, Waltham, MA, USA).

For physiological and activity measurements, cells were harvested from PA growth conditions and after 24 h–72 h of growth in respective experimental conditions. Before activity measurements, cells were resuspended in fresh BG11 medium at the desired Chl concentration and acclimated under the respective growth conditions before the measurements. For protein extraction, cells were harvested from PA growth conditions and after 72 h of growth under photomixotrophic conditions. For next‐generation sequencing, cells were grown six generations under PA conditions prior to the cell harvesting.

### Membrane inlet mass spectrometry (MIMS)

Membrane inlet mass spectrometry (MIMS) measurements were performed as described by Ermakova et al. (2016). Cells were collected, centrifuged, and resuspended in the respective fresh medium (pH 7.5) to a final concentration of 15 μg Chl L^−1^. ^18^O‐enriched O_2_ (98% ^18^O_2_; CK Isotopes) was bubbled into the suspension until approximately equal concentrations of ^16^O_2_ and ^18^O_2_ were reached. Gas exchange rates were determined according to Beckmann et al. (2009). The O_2_ and CO_2_ exchange measurements were monitored during the initial 5 min dark period, followed by 5 min of high light illumination (500 μmol photons m^−2^ s^−1^), and a subsequent 5 min dark phase. A second measurement cycle was then performed in which O_2_ exchange was recorded during 5 min of high light illumination (500 μmol photons m^−2^ s^−1^) in the presence of 500 μM DCBQ or 500 μm DMBQ (added immediately before the light phase) and finally followed by a 3 min dark period. Q_A_ reoxidation measurements.

The kinetics of the Chl fluorescence decay after a single‐turnover saturating flash was monitored using a fluorometer (FL 3500; PSI Instruments) according to Vass et al. (1999). Cells were adjusted to a Chl concentration of 5.0 μg ml^−1^ and dark‐adapted for 5 min before measurements. When indicated, measurements were performed in the presence of 20 μm DCMU. In some experiments, cells were illuminated for 30 s with a strong FR light (720 nm, 75 W m^−2^) prior to saturating flash. Fitting of fluorescence relaxation was done according to Vass et al. (1999); two exponential (for the fast and middle phases) and a hyperbolic function for the slow phase that arises from charge recombination were applied. When measurements were performed with DCMU, one exponential (for the very small fast phase) and one hyperbolic (for the slow phase) function were used.

### Photosynthetic yield by Chl fluorescence and P700 absorption

The Chl fluorescence and the P700 signal from intact cells were recorded with a pulse amplitude‐modulated fluorometer (Dual‐PAM‐100; Walz, Effeltrich, Germany) as described by Huokko et al. (2017). Before measurements, cell suspensions at a Chl concentration of 15 μg ml^−1^ were dark‐adapted for 10 min. Saturating pulses of 5000 μmol photons m^−2^ s^−1^ (300 ms) were applied to samples when required. The effective yield of PSII, Y(II), was calculated as (*F*_m′_ − *F*_s_)/*F*_m′_ where the maximal fluorescence during illumination (*F*_m′_) was obtained from a saturating pulse applied after 100 s of illumination with actinic light (50 μmol photons m^−2^ s^−1^). The maximal P700 oxidation (Pm) level was induced by a SP applied on top of strong FR light (720 nm, 75 W m^−2^) toward the end of FR period. The effective yield of PSI [Y(I)] was calculated after 100 s of actinic illumination using the formula *Y*(*I*) = (Pm′ − *P*)/Pm, where Pm′ represents Pm during illumination.

### Thylakoid membrane extraction, protein electrophoresis, and immunoblot analysis

The total protein extract of the *Synechocystis* cells and the membrane protein fraction separated from it were isolated according to Zhang et al. (2009) with the exception that cells were disrupted by Bullet Blender Storm (Next Advance, Troy, NY, USA) with 0.15 mm diameter zirconium beads (Biotop). Total proteins (30 μg of total protein per sample) were separated by 12% (w/v) SDS‐PAGE containing 6 m urea, transferred to a polyvinylidene difluoride membrane (Immobilon‐P; Merck Millipore, Burlington, MA, USA), and examined with a protein‐specific antibody against Psb28‐2 (Boehm et al., 2012) in dilution of 1:2000. A secondary antibody (AS09 602, Goat anti‐Rabbit IgG (H&L), HRP‐conjugated, Agrisera, Vännäs, Sweden) was applied in a dilution of 1:20000, and immunoblots were exposed to X‐ray films (Fuji, Tokyo, Japan).

Protein complexes in their native form in isolated membrane fractions of *Synechocystis* were studied by blue native (BN)‐PAGE according to Zhang et al. (2004). About 150 μg of proteins from the thylakoid membrane fraction per sample were loaded to BN‐PAGE gels. For separation of proteins in the second dimension, the lanes of the BN gel were excised and incubated in SDS sample buffer according to Zhang et al. (2004) to solubilize proteins. The lane was then laid onto a 1‐mm‐thick 12% SDS‐PAGE gel with 6 M urea. After electrophoresis, proteins were transferred to polyvinylidene difluoride membrane (Immobilon‐P; Millipore) and detected with specific antibodies against PsbA (AS11 1786, Agrisera) in dilution of 1:5000 and against Psb28‐2 in dilution of 1:2000 (Boehm et al., 2012). Secondary antibody (AS09 602, Goat anti‐Rabbit IgG (H&L), HRP‐conjugated, Agrisera) was applied in dilution of 1:20000 and immunoblots were exposed to X‐ray films (Fuji).

### Proteomics sample preparation

Total protein extract was isolated as described above, after which protein concentrations in the lysates were measured using a Bradford assay (Bio‐Rad (Hercules, CA, USA) protein assay dye reagent) using BSA as a standard at concentrations of 0, 0.05, 0.1, 0.2, and 0.4 mg ml^−1^. Lysates were diluted to a protein concentration of 1 mg ml^−1^ before being reduced with 10 mm DTT for 45 min at 55°C. Samples were then alkylated with 17 mm IAA for 30 min at 25°C before being digested by a trypsin/LysC protease mix (Pierce Trypsin/LysC protease mix, MS grade, Thermo Scientific) at a protein to protease mass ratio of 50:1 for 16 h at 37 °C with 600 rpm of shaking. Samples were then quenched with formic acid to pH <2 and desalted through stage tips packed with six layers of C18 matrix using the following chromatographic workflow: activation with 50 μL acetonitrile, equilibration with 200 μL 0.1% formic acid, sample application, two times wash with 200 μL 0.1% formic acid and two times elution with 30 μL 80% acetonitrile, 0.1% formic acid. Samples were then evaporated to dryness at 40°C in a speedvac before being resuspended in 20 μL 0.1% formic acid and stored at −20°C until mass spectrometry analysis.

### Proteomics mass spectrometry analysis

Proteomics analysis was performed on a Q‐exactive HF Hybrid Quadrupole‐Orbitrap Mass Spectrometer coupled with an UltiMate 3000 RSLCnano System with an EASY‐Spray ion source. About 2 μL sample was loaded onto a C18 Acclaim PepMap 100 trap column (75 μm × 2 cm, 3 μm, 100 Å) with a flow rate of 7 μL per min, using 3% acetonitrile, 0.1% formic acid, and 96.9% water as solvent. The samples were then separated on an ES802 EASY‐Spray PepMap RSLC C18 Column (75 μm × 25 cm, 2 μm, 100 Å) with a flow rate of 3.6 μL per minute for 40 min using a linear gradient from 1% to 32% with 95% acetonitrile, 0.1% formic acid and 4.9% water as secondary solvent. Mass spectrometry analysis was performed using one full scan (resolution 30 000 at 200 m/z, mass range 300–1200 m/z) followed by 30 MS2 DIA scans (resolution 30 000 at 200 m/z, mass range 350–1000 m/z) with an isolation window of 10 m/z. The maximum injection times for the MS1 and MS2 were 105 and 55 ms, respectively, and the automatic gain control was set to 3·106 and 1·106, respectively. Precursor ion fragmentation was performed with high‐energy collision‐induced dissociation at an NCE of 26 for all samples.

The prosit intensity prediction model “Prosit_2020_intensity_hcd” was used to generate a predicted peptide library from a FASTA file of the UniProt proteome set Synechocystis sp. PCC 6803: UP000001425.

The raw spectra were converted to mzML using MSconvert and then searched using the EncyclopeDIA v. 1.2.2 search engine. Peptides detected in at least three replicates in every sample group were tested for differential peptide abundance using the MSstats package (version 4.12.0) in R (version 4.3.1.). For every peptide in each comparison MSstats estimated fold changes and *P*‐values adjusted for multiple hypothesis testing (Benjamini–Hochberg method) with a significance threshold of 0.01.

### RNA extraction and reverse transcription‐quantitative PCR (RT‐qPCR)

About 30 ml of cell culture was harvested at a designated time under auto‐ and photomixotrophic conditions. The sample was then centrifuged at 6000 rpm for 10 min, flash‐frozen in liquid nitrogen, and stored at −20°C before extraction. RNA was isolated using the TRIzol® Reagent (Invitrogen, Carlsbad, CA, USA), treated with TURBO DNA‐free™ Kit (Invitrogen, Carlsbad, CA, USA), and purified with the chloroform:phenol:isoamyl alcohol method. cDNA was synthesized using the iScript cDNA Synthesis kit (Bio‐Rad, Hercules, CA, USA) with random primers. The qPCR reaction was carried out in 10 μL reactions composed of 5 μL of iQ SYBR® Green Supermix (Bio‐Rad), 2 μL dH_2_O, 2 μL diluted cDNA template (15 ng of RNA), and 0.5 μL mix of each 10 μm forward and reverse PCR primer, utilizing the Bio‐Rad IQ5 system using *rrn16Sa* as a reference gene. Two technical replicates and three biological replicates were performed using the *psb28‐1*, *psb28‐2*, and *rrn16Sa* primers (Table S4). WT 1 at 0 time point for each gene was selected as a calibrator condition, and the fold change (2^−ΔΔCt^) was calculated.

### Sequence alignment of Psb28‐2 and Psb28‐1

The amino acid sequences of Psb28‐1 and Psb28‐2 were retrieved from UniProt (accession numbers Q55356 and P73731, respectively) and aligned using the ClustalX program (https://www.genome.jp/tools‐bin/clustalw) with default parameters. The resulting alignments were visualized and corrected in JalView (Waterhouse et al., 2009) and UCSF ChimeraX (Meng et al., 2023) was used to visualize structural alignment.

### Genomic DNA isolation, next‐generation sequencing (NGS), and constructing a phylogenetic tree

Genomic DNA from WT strains was isolated using the hot‐phenol extraction method (Williams, 1988), and sent for next‐generation sequencing at Macrogen Europe B.V. (Netherlands). Sequencing reads from WT strains 1–4 were mapped to the *Synechocystis* sp. PCC 6803 reference genome (Kaneko et al., 1996) using BWA‐MEM (v0.7.17‐r1188) (Li and Durbin, 2009) with default parameters, including a minimum seed length of 19, a maximum gap length of 100, and standard mismatch and gap penalties. SAM files were converted to BAM format using GATK SamFormatConverter (v4.2.5.0), sorted with GATK SortSam, and indexed with GATK BuildBamIndex, all using default parameters. Variant calling was performed using BCFTools (v1.10.2) (Li, 2011) with the mpileup function, which calculates genotype likelihoods, and the call function, which identifies variants with the default method. Variants were annotated using SnpEff (v5.1) (Cingolani et al., 2012), referencing the built‐in *Synechocystis* sp. PCC 6803 database, with splice site and upstream/downstream regions limited to 100 bp. Variants with Phred quality scores <30 were excluded using SnpSift (v5.1) with the filter string QUAL ≥30.

FASTA files were generated from variant calls using BCFTools norm and consensus functions with default parameters. The phylogenetic tree was constructed with BEAST (Suchard et al., 2018) using default settings, and the concordance graph was created with the UpSetR package (Conway et al., 2017) and visualized using FigTree (http://tree.bio.ed.ac.uk/software/figtree/).

### Glucose determination

Glucose concentration in the growth media was determined spectrophotometrically with the commercial Megazyme Sucrose/D‐Glucose Assay Kit (NEOGEN) according to the manufacturer's instructions.

### Glycogen extraction and determination

Intracellular glycogen content was extracted and determined according to Ortega‐Martínez et al. (2023).

## AUTHOR CONTRIBUTIONS

YA conceptualized the study and designed the research, TH designed the research, performed the majority of the experiments, analyzed the data and wrote the first draft of the manuscript, ES conducted mass spectrometry analyses, BK analyzed and annotated genome sequencing results, PN isolated RNA and performed RT‐PCR, PPP fitted fluorescence decay kinetics, LW supervised genome sequence analysis, performed DKN measurements and analyzed corresponding data, MH isolated genomic DNA for sequencing, LN performed part of MIMS experiments and contributed to data analysis, NK carried out sequence alignment, OV performed some growth experiments and flash fluorescence measurements for verification of earlier obtained data. JK, PH, and IV provided essential resources and were actively involved in revising the manuscript throughout its preparation. TH and YA finalized the manuscript with contributions from all authors. All authors have read and approved the final version of the manuscript.

## Supporting information


**Figure S1.** (a) The cell number per OD_750_ in WT strains grown 72h under photomixotrophy. (b–d) The growth of WT strains under (b) photoautotrophy (PA), (c) photomixotrophy, BG‐11 medium adjusted to pH 7.5 at inoculation (PM pH 7.5) without extra bicarbonate supplementation and (d) photomixotrophy, BG‐11 medium adjusted to pH 8.2 at inoculation (PM pH 8.2) monitored by OD_750_. (e) The ratio of linear electron transfer (LET) to cyclic electron transfer (CET) after 72h of photomixotrophy quantified by measuring DIRK of the P700 and Pc signals using the DKN, deducted from KNS. (f) Initial signal decay of the deconvoluted P700 and plastocyanin (PC) signals during dark interval relaxation kinetics (DIRK) measurements, performed in the presence and absence of DCMU supplementation. These measurements were used to calculate PSI electron transfer rates, which were subsequently utilized to determine the ratio of LET to CET in panel (e). (g, h) The redox kinetics of (g) P700 and (h) Fd in WTs 1 and 3 after 72 hours of growth under PM. In (g) and (h) grey bar = darkness, red bar = red actinic light (AL) illumination (3400 μmol photons m^−2^ sec^−1^), burgundy bar = far red‐light (FR) illumination, arrow = a saturating pulse (SP; 5000 μmol photons m^−2^ sec^−1^, 50 ms). In (a) values are means ± SD; *n* = 9 cell counts from 3 biological replicates with individual data points shown as circles, in (b–d) values are means ± SD; *n* = 3 biological replicates, and in (e, f) values are means ± SD; *n* = 2–3 biological replicates, in (g‐h) data is shown as means ± SEM; *n* = 3.
**Figure S2.** Kinetics of O_2_ uptake rate in WTs 1 and 2 grown under (a, c) photoauto‐ (PA) and 72 h under (b,d) photomixotrophy (PM).
**Figure S3.** Kinetics of gross O_2_ production in (a) WT1 and (b) WT2 grown photoautotrophically (PA) or for 72h under photomixotrophy (PM), measured in the presence or absence of the artificial electron acceptors DCBQ and DMBQ.
**Figure S4.** Kinetics of CO_2_ exchange rate in WTs 1 and 2 grown under (a,c) photoauto‐ (PA) and (b, d) 72 h under photomixotrophy (PM).
**Figure S5.** Relaxation of flash‐induced fluorescence yield in WT cells grown (a) under photoautotrophic conditions (PA) and under photomixotrophy (PM) for (b) 24h and (c) 48h.
**Figure S6.** Relaxation of flash‐induced fluorescence yield after (a) photoautotrophic (PA) growth, and after (b) 24 h and (c) 48 h of photomixotrophic (PM) growth in the presence of DCMU.
**Figure S7.** Relaxation of flash‐induced fluorescence yield in WTs 1 and 2 grown (a) 24h and (b) 48h under photomixotrophy with and without pre‐illumination with far‐red (FR) light.
**Figure S8.** Confirmation of amplicon sizes for the selected genes studied in WTs 1 and 3.
**Figure S9.** Quantitative reverse transcription (RT‐q) PCR analysis of *psb28‐1* transcript abundancies in WTs 1 and 3 under photomixotrophic conditions normalized to *rrn 16Sa* at the designated time points.
**Figure S10.** Relative quantification of Psb28‐2 association with various forms of PSII under photoautotrophic (PA) and 72 h under photomixotrophic (PM) conditions in WTs 1 and 3, based on 2D‐BN‐PAGE immunoblotted with antibody against Psb28‐2 (Figure 4d,e).
**Table S1.** Proteins identified and quantified at least with two peptides in WTs 1 and 3 using global label‐free MS/MS with data‐independent acquisition (DIA), *P* ≤ 0.05.
**Table S2.** Proteins upregulated in WT 1 compared with WT 3 under 72 h photomixotrophic conditions (fold change (FC) ≥1.5, *P* ≤ 0.05).
**Table S3.** Proteins downregulated in WT 1 compared with WT 3 under 72 h photomixotrophic conditions (fold change (FC) ≤−1.5, *P* ≤ 0.05).
**Table S5.** All types of mutations identified in WT 1 compared with the WT Kazusa reference strain.
**Table S6.** All types of mutations identified in WT 2 compared with the WT Kazusa reference strain.
**Table S7.** All types of mutations identified in WT 3 compared with the WT Kazusa reference strain.
**Table S8.** All types of mutations identified in WT 4 compared with the WT Kazusa reference strain.
**Table S9.** Mutations in coding regions resulting in frameshifts, missense, or premature stop codons across studied WT strains, including shared and unique mutated gene.

## Data Availability

The genome sequence data have been deposited in the National Center for Biotechnology Information (NCBI) under BioProject accession PRJNA1277488 (https://www.ncbi.nlm.nih.gov/bioproject/?term=PRJNA1277488), BioSample accession numbers SAMN49110441, SAMN49110442, SAMN49110443, SAMN49110444, SAMN49110445, SAMN49110446, and SAMN49110447 (https://www.ncbi.nlm.nih.gov/biosample?LinkName=bioproject_biosample_all&from_uid=1277488) and SRA numbers SRR33998957, SRR33998958, SRR33998959, SRR33998960, SRR33998961, SRR33998962, and SRR33998963 (https://www.ncbi.nlm.nih.gov/sra/?term=PRJNA1277488). The mass spectrometry proteomics data have been deposited to the ProteomeXchange Consortium via the PRIDE partner repository with the dataset identifier PXD066779 (https://www.ebi.ac.uk/pride/archive/projects/PXD066779). The data that support the findings of this study are available in the supplementary material of this article. The datasets analyzed during the current study are available from the corresponding author upon reasonable request.
