# Supplementary material for: Differences in photosystem II activity and carbon allocation during photomixotrophic growth in distinct wild‐type strains of Synechocystis sp. PCC 6803
Source: Plant J. 2026 Jan 24;125(2):e70683. doi: 10.1111/tpj.70683 (PMC12831587; doi:10.1111/tpj.70683)
Supplement: Supplementary file 1 — Figure S1. (a) The cell number per OD750 in WT strains grown 72h under photomixotrophy. (b–d) The growth of WT strains under (b) photoautotrophy (PA), (c) photomixotrophy, BG‐11 medium adjusted to pH 7.5 at inoculation (PM pH 7.5) without extra bicarbonate supplementation and (d) photomixotrophy, BG‐11 medium adjusted to pH 8.2 at inoculation (PM pH 8.2) monitored by OD750. (e) The ratio of linear electron transfer (LET) to cyclic electron transfer (CET) after 72h of photomixotrophy quantified by measuring DIRK of the P700 and Pc signals using the DKN, deducted from KNS. (f) Initial signal decay of the deconvoluted P700 and plastocyanin (PC) signals during dark interval relaxation kinetics (DIRK) measurements, performed in the presence and absence of DCMU supplementation. These measurements were used to calculate PSI electron transfer rates, which were subsequently utilized to determine the ratio of LET to CET in panel (e). (g, h) The redox kinetics of (g) P700 and (h) Fd in WTs 1 and 3 after 72 hours of growth under PM. In (g) and (h) grey bar = darkness, red bar = red actinic light (AL) illumination (3400 μmol photons m−2 sec−1), burgundy bar = far red‐light (FR) illumination, arrow = a saturating pulse (SP; 5000 μmol photons m−2 sec−1, 50 ms). In (a) values are means ± SD; n = 9 cell counts from 3 biological replicates with individual data points shown as circles, in (b–d) values are means ± SD; n = 3 biological replicates, and in (e, f) values are means ± SD; n = 2–3 biological replicates, in (g‐h) data is shown as means ± SEM; n = 3. Figure S2. Kinetics of O2 uptake rate in WTs 1 and 2 grown under (a, c) photoauto‐ (PA) and 72 h under (b,d) photomixotrophy (PM). Figure S3. Kinetics of gross O2 production in (a) WT1 and (b) WT2 grown photoautotrophically (PA) or for 72h under photomixotrophy (PM), measured in the presence or absence of the artificial electron acceptors DCBQ and DMBQ. Figure S4. Kinetics of CO2 exchange rate in WTs 1 and 2 grown under (a,c) photo [file TPJ-125-0-s001.zip › tpj70683-sup-0001-Supinfo.docx]

**Supplementary Information**

**Differences in Photosystem II activity and carbon allocation during photomixotrophic growth in distinct wild-type strains of Synechocystis sp. PCC 6803**

**Tuomas Huokko^1^, Emil Sporre^2^, Bradley Koch^3^**, **Priyanka Pradeep Patil^4,5^, Laura Wey^1^, Lauri Nikkanen^1^_,_ Pornpan Napaumpaiporn^1^_,_ Olli Virtanen^1^, Michal Hubacek^1^, Natalia Kulik^6^, Josef Komenda^6^, Elton Hudson^2^, Imre Vass^4^, Yagut Allahverdiyeva^1*^**

^1^ Molecular Plant Biology, Department of Life Technologies, University of Turku, Finland.

^2^ Department of Protein Science, Science for Life Laboratory, KTH- Royal Institute of Technology, Stockholm, Sweden

^3^ University of Helsinki, Finland.

^4^ Institute of Plant Biology, HUN-REN Biological Research Center of HAS, Hungary.

^5^ Doctoral School of Biology, Faculty of Science and Informatics, University of Szeged, Szeged, Hungary

^6^ Institute of Microbiology, Centre Algatech, Czech Republic

* corresponding author: Yagut Allahverdiyeva

**Email:**  [allahve@utu.fi](mailto:allahve@utu.fi)


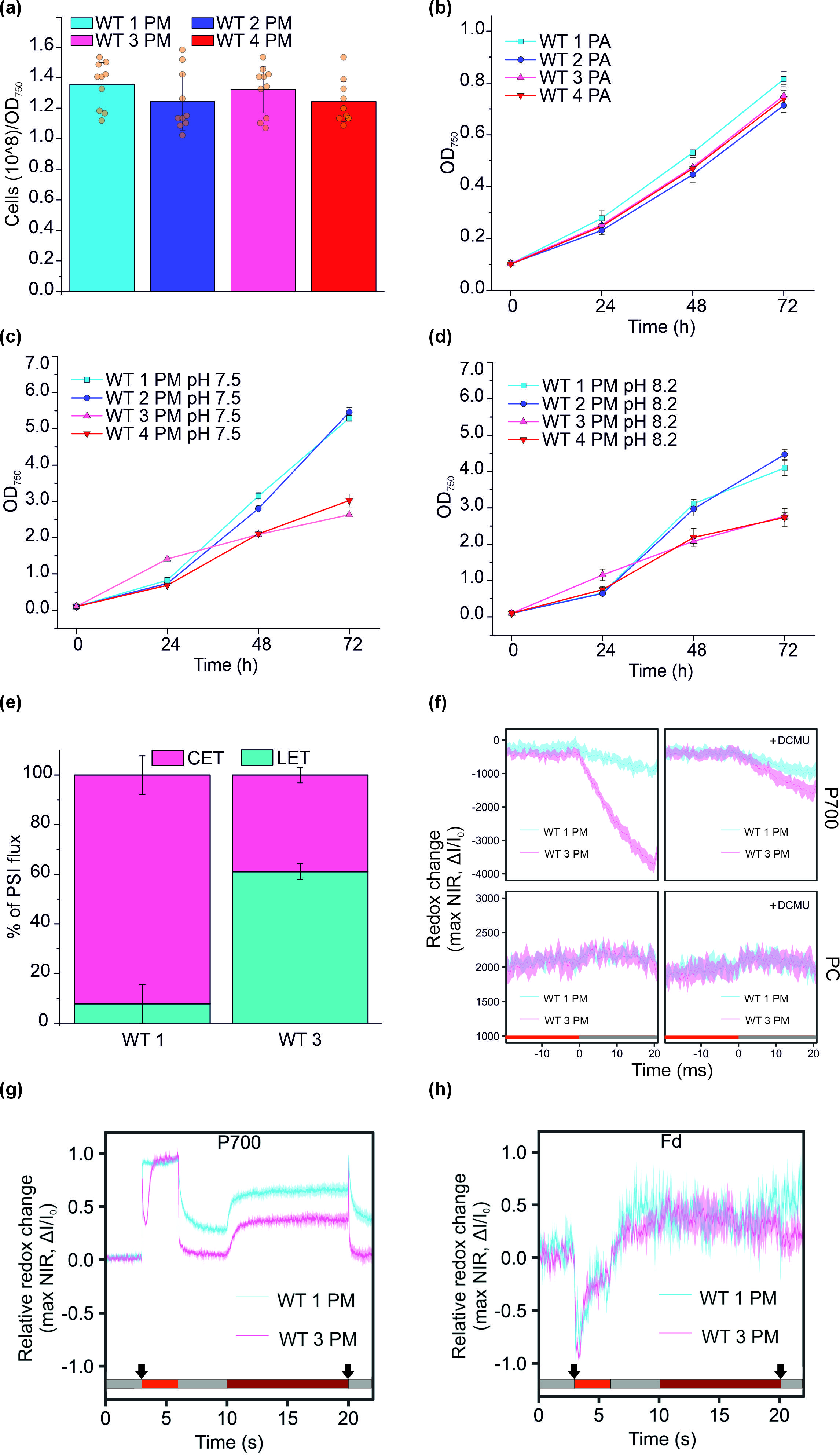


**Figure S1.** (a) The cell number per OD_750_ in WT strains grown 72h under photomixotrophy. (b-d) The growth of WT strains under (b) photoautotrophy (PA), (c) photomixotrophy, BG-11 medium adjusted to pH 7.5 at inoculation (PM pH 7.5) without extra bicarbonate supplementation and (d) photomixotrophy, BG-11 medium adjusted to pH 8.2 at inoculation (PM pH 8.2) monitored by OD_750_. (e) The ratio of linear electron transfer (LET) to cyclic electron transfer (CET) after 72h of photomixotrophy quantified by measuring DIRK of the P700 and Pc signals using the DKN, deducted from KNS. (f) Initial signal decay of the deconvoluted P700 and plastocyanin (PC) signals during dark interval relaxation kinetics (DIRK) measurements, performed in the presence and absence of DCMU supplementation. These measurements were used to calculate PSI electron transfer rates, which were subsequently utilized to determine the ratio of LET to CET in panel (e). (g,h) The redox kinetics of (g) P700 and (h) Fd in WTs 1 and 3 after 72 hours of growth under PM. In (g) and (h) grey bar= darkness, red bar= red actinic light (AL) illumination (3400 μmol photons m^−2^ sec^−1^), burgundy bar= far red-light (FR) illumination, arrow= a saturating pulse (SP; 5000 μmol photons m^−2^ sec^−1^, 50 ms). In (a) values are means ± SD; n = 9 cell counts from 3 biological replicates with individual data points shown as circles, in (b-d) values are means ± SD; n = 3 biological replicates, and in (e-f) values are means ± SD; n = 2-3 biological replicates, in (g-h) data is shown as means ± SEM; n = 3.


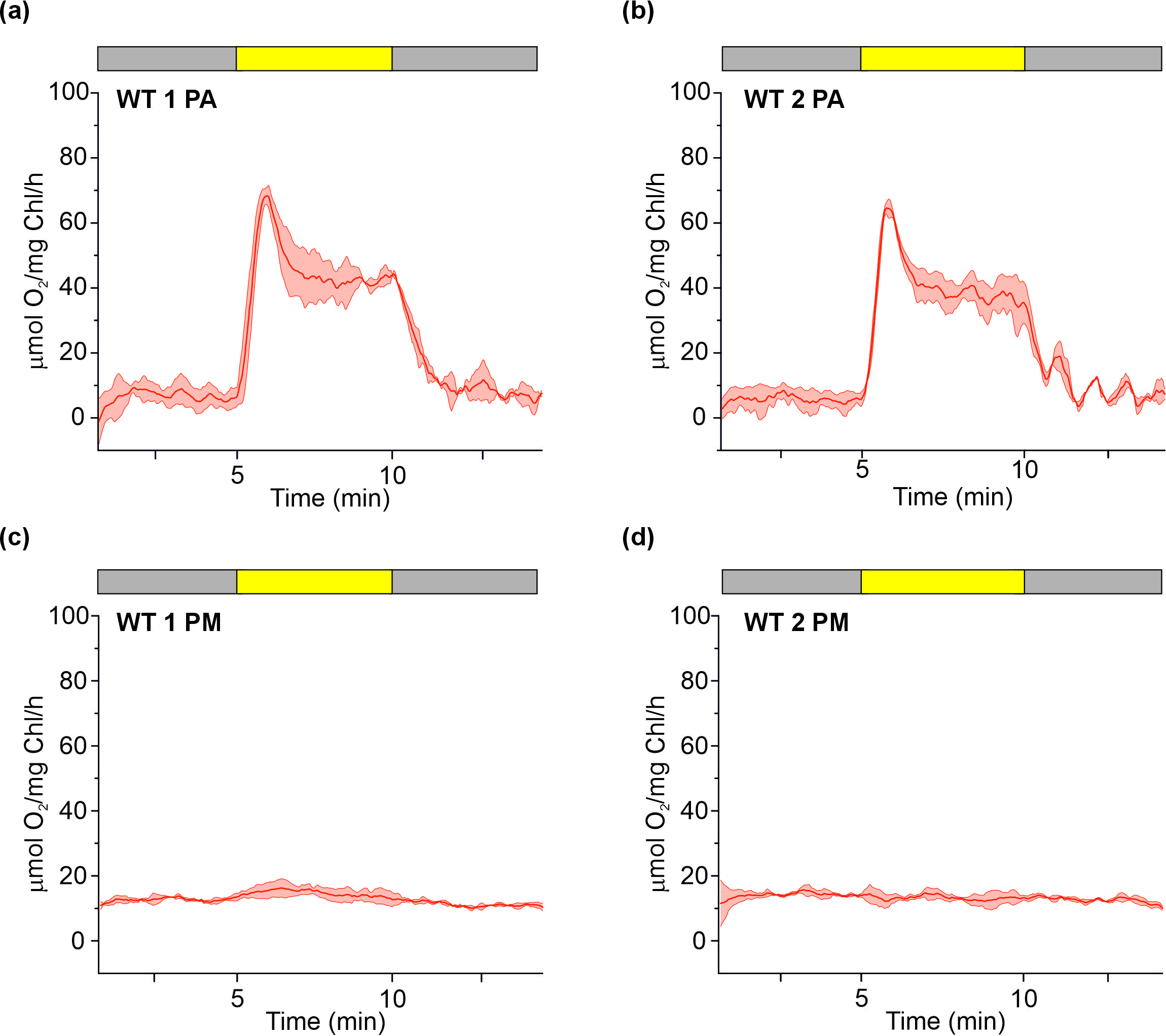


**Figure S2.** Kinetics of O_2_ uptake rate in WTs 1 and 2 grown under (a,c) photoauto- (PA) and 72h under (b,d) photomixotrophy (PM). Grey bar=darkness, yellow bar=illumination with 500 µmol photons m^-2^ s^-1^. Values are means ± SD; n = 3-4 biological replicates.


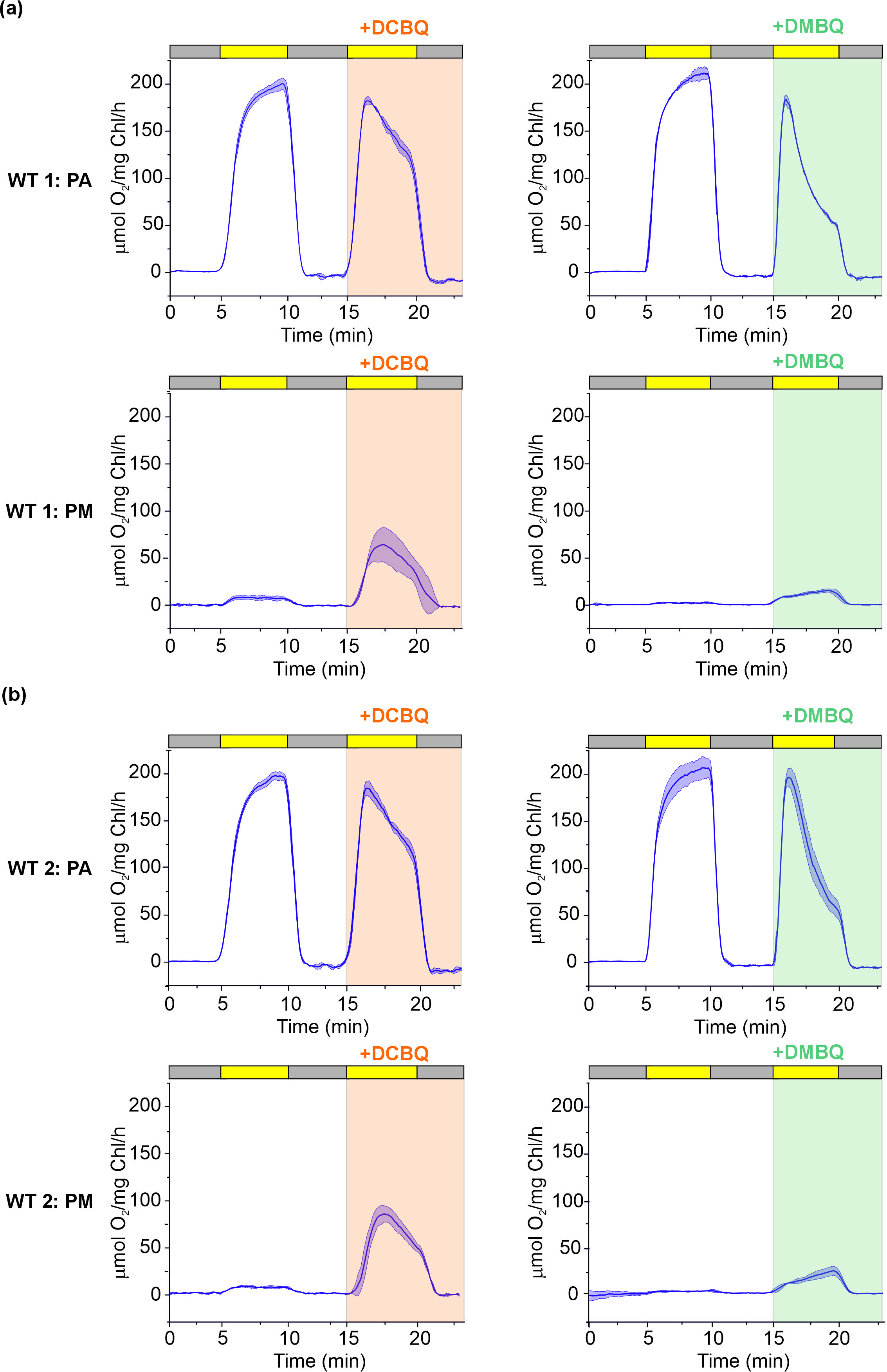


**Figure S3.** Kinetics of gross O_2_ production in (a) WT1 and (b) WT2 grown photoautotrophically (PA) or for 72h under photomixotrophy (PM), measured in the presence or absence of the artificial electron acceptors DCBQ and DMBQ. Grey bar=darkness, yellow bar=illumination with 500 µmol photons m^-2^ s^-1^. Orange shading indicates measurement phases with DCBQ, and light green shading phases with DMBQ. Values are means ± SD; n = 3-4 biological replicates.


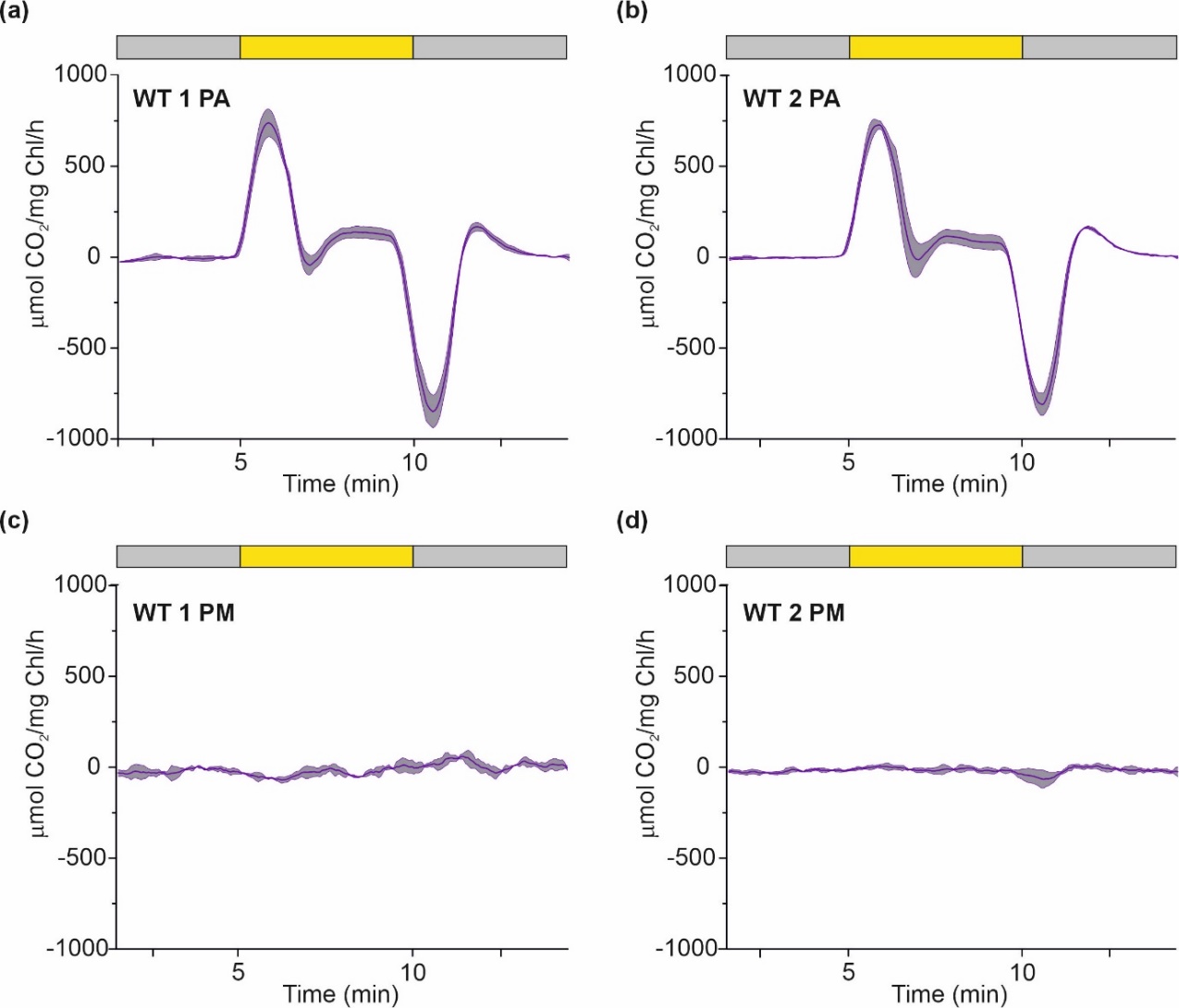


**Supplementary Figure 4.** Kinetics of CO_2_ exchange rate in WTs 1 and 2 grown under (a,c) photoauto- (PA) and (b,d) 72h under photomixotrophy (PM). Grey bar=darkness, yellow bar=illumination with 500 µmol photons m^-2^ s^-1^. Values are means ± SD; n = 3-4 biological replicates.


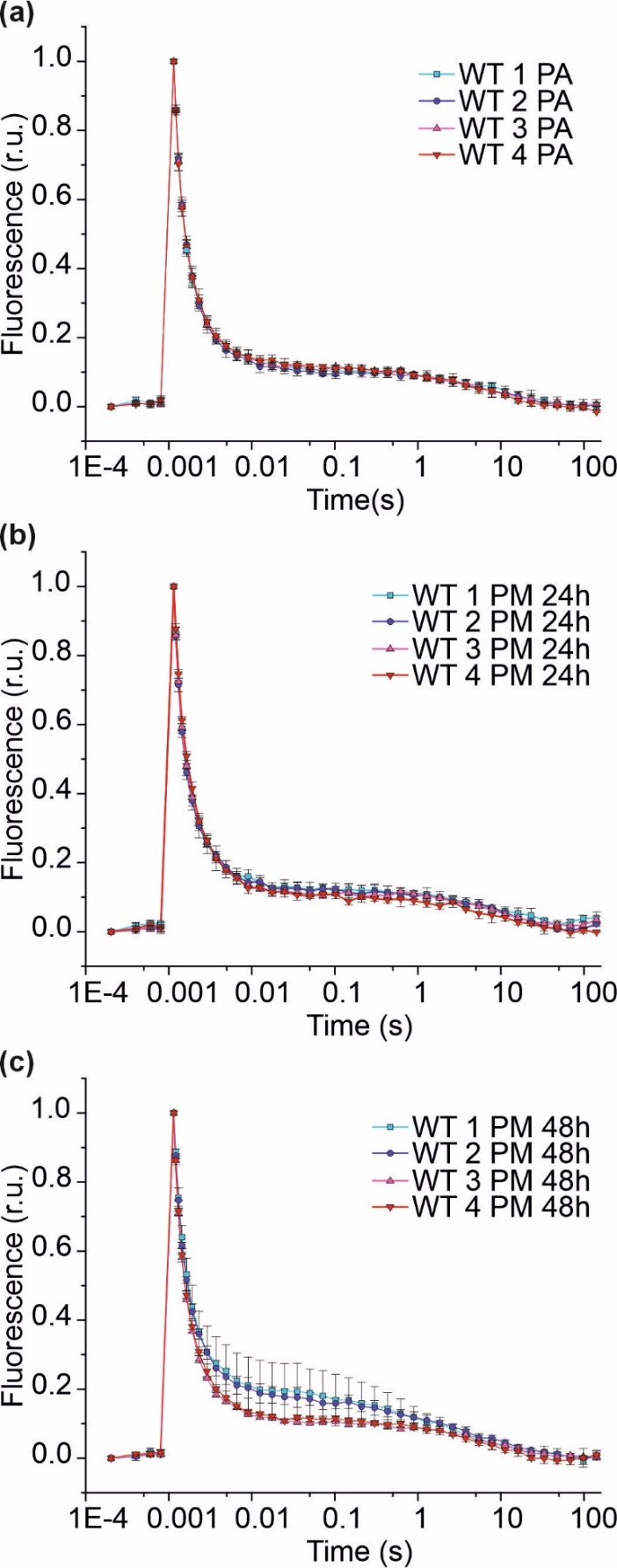


**Figure S5.** Relaxation of flash-induced fluorescence yield in WT cells grown (a) under photoautotrophic conditions (PA) and under photomixotrophy (PM) for (b) 24h and (c) 48h. Values are means ± SD; n = 3 biological replicates.


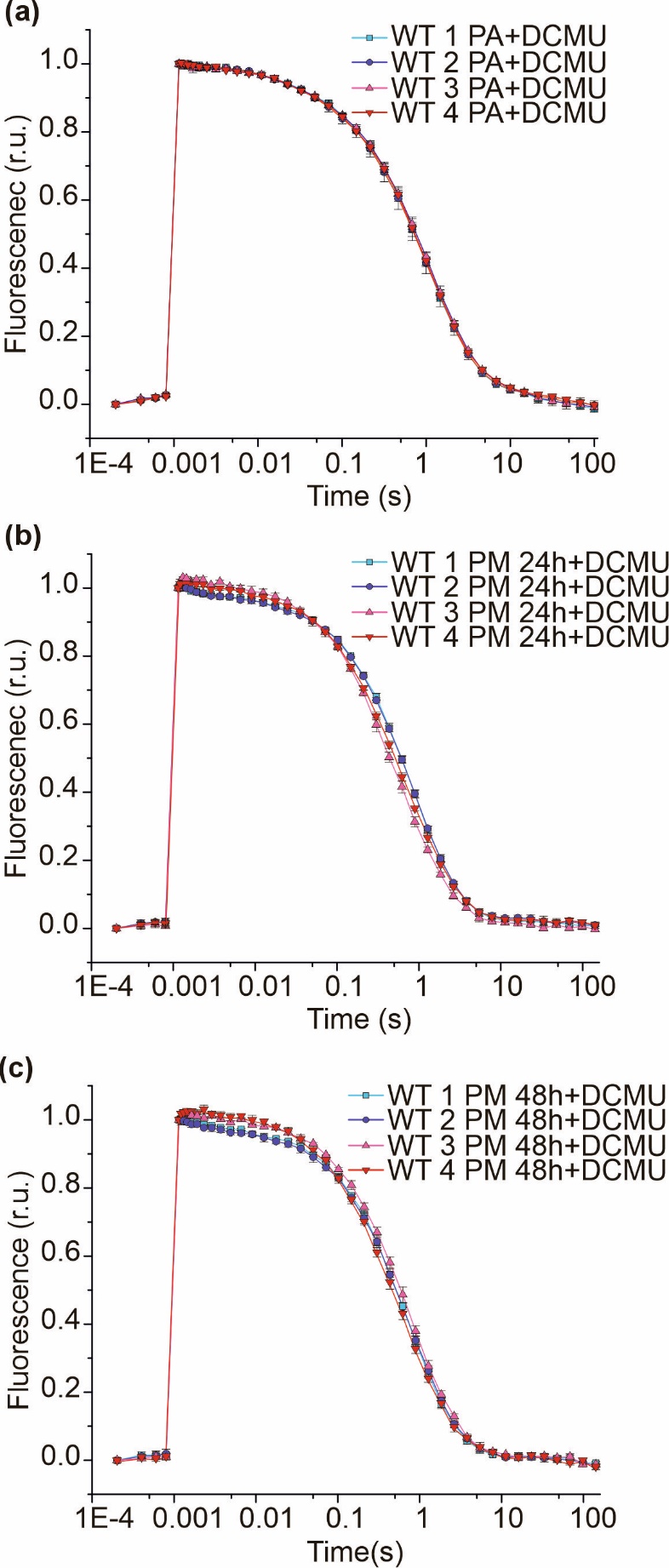


**Figure S6.** Relaxation of flash-induced fluorescence yield after (a) photoautotrophic (PA) growth, and after (b) 24 h and (c) 48 h of photomixotrophic (PM) growth in the presence of DCMU. Data represent means ± SD; n = 3 biological replicates.


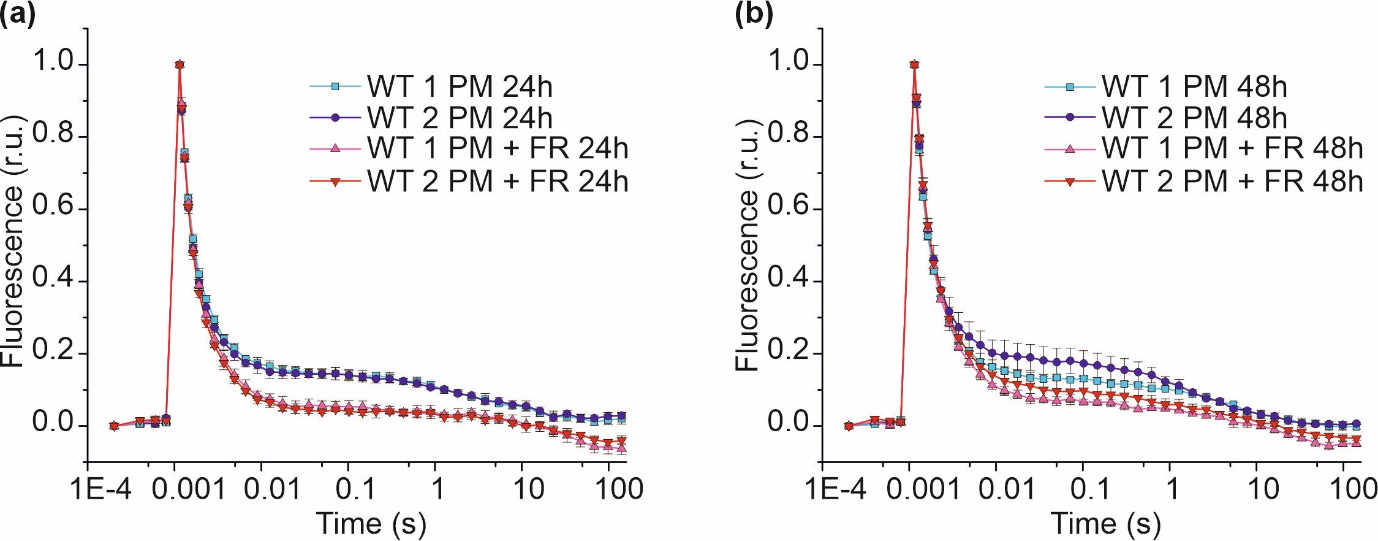


**Figure S7.** Relaxation of flash-induced fluorescence yield in WTs 1 and 2 grown (a) 24h and (b) 48h under photomixotrophy with and without pre-illumination with far-red (FR) light. Values are means ± SD; n = 3 biological replicates.


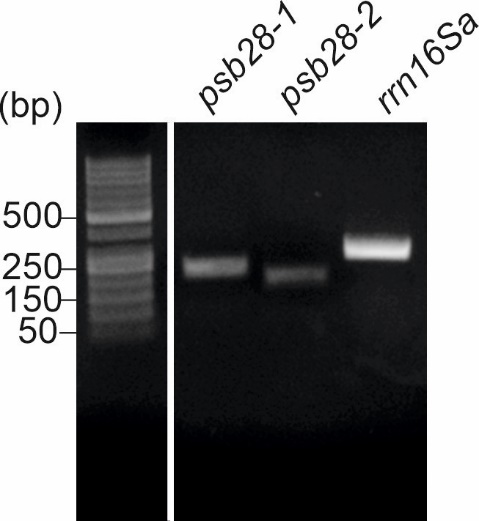


**Figure S8**. Confirmation of amplicon sizes for the selected genes studied in WTs 1 and 3. Agarose gel electrophoresis showing specific PCR products of the expected sizes for each gene.


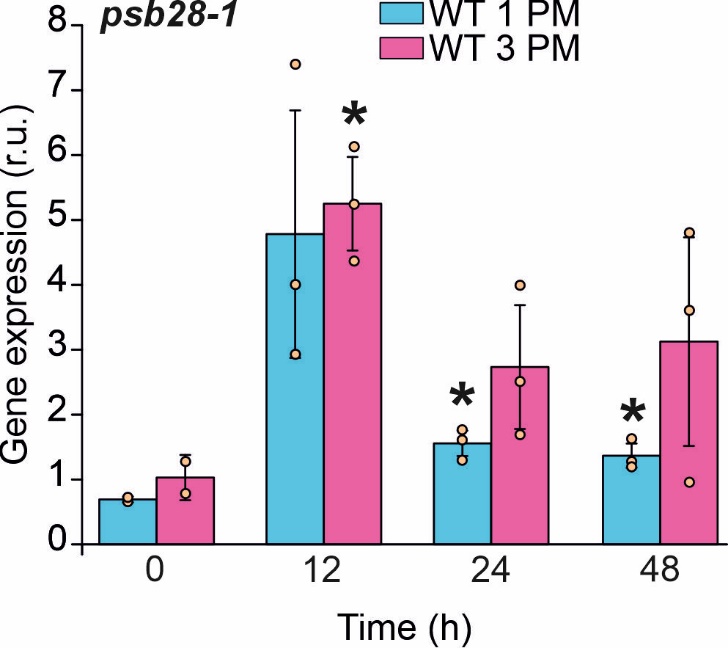


**Figure S9**. Quantitative reverse transcription (RT-q) PCR analysis of *psb28-1* transcript abundancies in WTs 1 and 3 under photomixotrophic conditions normalized to *rrn 16Sa* at the designated time points. Values are means ± SD; n = 3-5 biological replicates. Asterisk indicates statistically significant difference (*, P < 0.05) compared to timepoint 0h, according to unpaired Student's t-test.


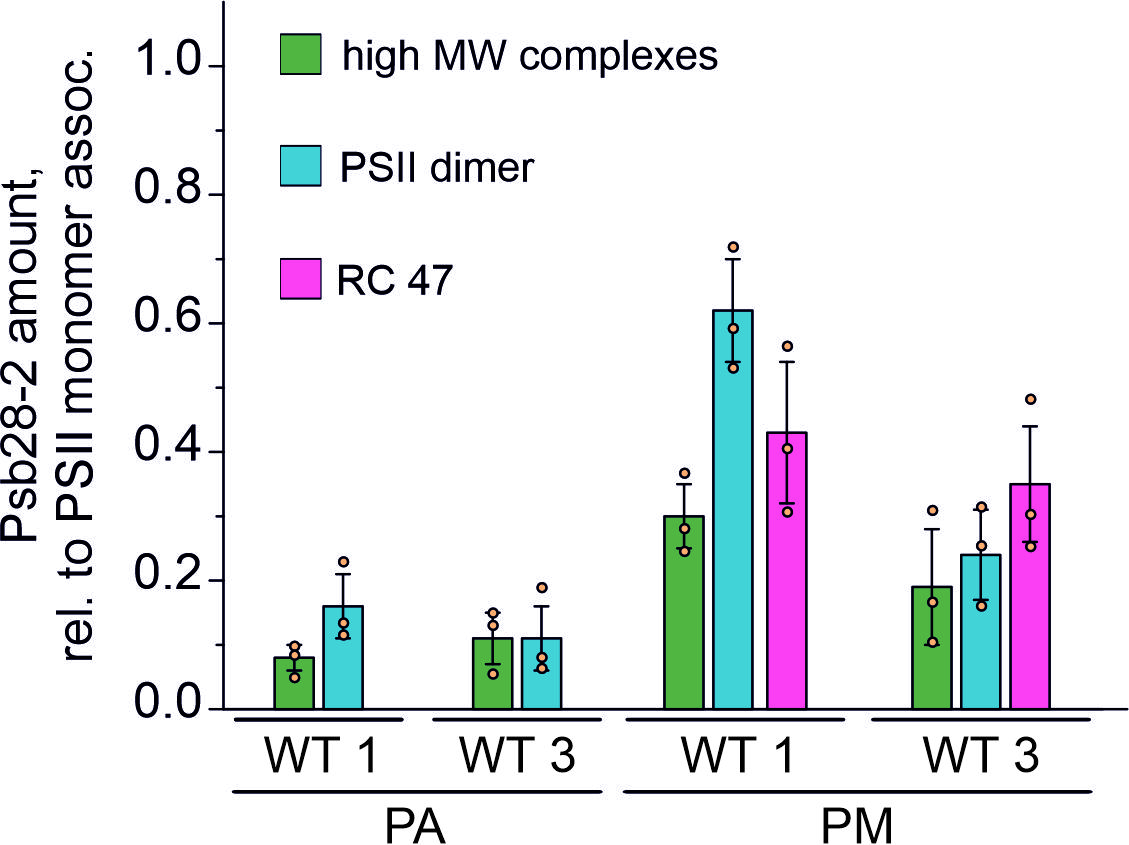


**Figure S10**. Relative quantification of Psb28-2 association with various forms of PSII under photoautotrophic (PA) and 72 h under photomixotrophic (PM) conditions in WTs 1 and 3, based on 2D-BN-PAGE immunoblotted with antibody against Psb28-2 (Figs. 4d and e). Values are means ± SD; n = 3 biological replicates with individual data points shown as circles.

**Supplementary Tables 1-3 and 5-9 are as separate Excel files**

**Table S1.** Proteins identified and quantified at least with two peptides in WTs 1 and 3 using global label-free MS/MS with data-independent acquisition (DIA), P ≤ 0.05.

**Table S2.** Proteins upregulated in WT 1 compared to WT 3 under 72 h photomixotrophic conditions (fold change (FC) ≥ 1.5, P ≤ 0.05).

**Table S3.** Proteins downregulated in WT 1 compared to WT 3 under 72 h photomixotrophic conditions (fold change (FC) ≤ −1.5, P ≤ 0.05).

**Table S5.** All types of mutations identified in WT 1 compared to the WT Kazusa reference strain.

**Table S6.** All types of mutations identified in WT 2 compared to the WT Kazusa reference strain.

**Table S7.** All types of mutations identified in WT 3 compared to the WT Kazusa reference strain.

**Table S8.** All types of mutations identified in WT 4 compared to the WT Kazusa reference strain.

**Table S9.** Mutations in coding regions resulting in frameshifts, missense, or premature stop codons across studied WT strains, including shared and unique mutated gene.

**Table S4**. Primer sequences and amplicon characteristics for RT-qPCR.

| **Oligoname** | **sequence** | **product size(bp)** | **Tm** | **Primer Efficiency (%)** | **Amplification factor (E)** |
| --- | --- | --- | --- | --- | --- |
| sll1398-psb28-1-F | gttgcagaaaccgttgtccc | 178 | 60 | 110.58 | 2.1 |
| sll1398-psb28-1-R | tgccctttacttccctggtg |  | 59.6 |  |  |
| slr1739-psb28-2-F | actcagcaatgtcagcctcc | 134 | 60 | 122.44 | 2.2 |
| slr1739-psb28-2-R | cagcaagacgtaaatggccg |  | 59.9 |  |  |
| rrn16Sa-F | gcggtaatacggaggatgca | 244 | 60 | 98.23 | 1.98 |
| rrn16Sa-R | gctttcgtccctcagtgtca |  | 60 |  |  |
|  |  |  |  |  |  |
|  |  |  |  |  |  |

**Supplementary Material and Methods**

**Cell Counting**

A Bürker counting chamber (Marienfeld-Superior) was applied to quantify the number of cells in a culture (normalized to OD_750_ = 1).

**Rates of linear electron transfer (LET) and cyclic electron transfer (CET) determined based on electron flux through PSI**

The rates of LET and CET were quantified by measuring dark interval relaxation kinetics (DIRK) of the P700 and plastocyanin (PC) signals using the DUAL-KLAS-NIR (Walz), as described by Theune et al. (2021). Cells were pre-illuminated for 2 min under 500 μmol photons m^−2^ s^−1^ after which the light was repeatedly (100x) shut off for 20 ms. The model spectra for *Synechocystis* were obtained from Nikkanen et al. (2020). The signals were averaged, and the electron flow through PSI was calculated as described by Theune et al (2021). The same sample was always measured in the presence and absence of 20 μM DCMU. Cells were adjusted to a Chl concentration of 15.0 µg mL^−1^.

**Assessing redox changes of P700 and Fd**

Redox changes of P700 and Fd were also resolved using a DUAL-KLAS-NIR (DKN) spectrophotometer (Walz) following the method of Nikkanen et al. (2020). Absorbance differences at 780–820, 820–870, 840–965, and 870–965 nm were used to deconvolute the redox states using a model plot developed for *Synechocystis*. Prior to measurements, samples were adjusted to 15 µg mL⁻¹ Chl *a* and dark-adapted for 15 minutes. Redox changes were tracked using a modified NIRMAX script involving a sequence of 3 s red actinic light (3400 µmol photons m⁻² s⁻¹), followed by a 4 s dark period and 10 s of far-red light. A saturating pulse (5500 µmol photons m⁻² s⁻¹, 50 ms) was applied 200 ms into the red-light period to fully reduce the Fd pool and at the end of FR phase to determine P_m_.
